# Stepwise Development of Hematopoietic Stem Cells from Embryonic Stem Cells

**DOI:** 10.1371/journal.pone.0004820

**Published:** 2009-03-16

**Authors:** Kenji Matsumoto, Takayuki Isagawa, Toshinobu Nishimura, Takunori Ogaeri, Koji Eto, Satsuki Miyazaki, Jun-ichi Miyazaki, Hiroyuki Aburatani, Hiromitsu Nakauchi, Hideo Ema

**Affiliations:** 1 Division of Stem Cell Therapy, Center for Stem Cell and Regenerative Medicine, Institute of Medical Science, University of Tokyo, Tokyo, Japan; 2 Genome Science Division, Research Center for Advanced Science and Technology, University of Tokyo, Tokyo, Japan; 3 Division of Stem Cell Regulation Research, Osaka University Graduate School of Medicine, Osaka, Japan; KU Leuven, Belgium

## Abstract

The cellular ontogeny of hematopoietic stem cells (HSCs) remains poorly understood because their isolation from and their identification in early developing small embryos are difficult. We attempted to dissect early developmental stages of HSCs using an *in vitro* mouse embryonic stem cell (ESC) differentiation system combined with inducible HOXB4 expression. Here we report the identification of pre-HSCs and an embryonic type of HSCs (embryonic HSCs) as intermediate cells between ESCs and HSCs. Both pre-HSCs and embryonic HSCs were isolated by their c-Kit^+^CD41^+^CD45^−^ phenotype. Pre-HSCs did not engraft in irradiated adult mice. After co-culture with OP9 stromal cells and conditional expression of HOXB4, pre-HSCs gave rise to embryonic HSCs capable of engraftment and long-term reconstitution in irradiated adult mice. Blast colony assays revealed that most hemangioblast activity was detected apart from the pre-HSC population, implying the early divergence of pre-HSCs from hemangioblasts. Gene expression profiling suggests that a particular set of transcripts closely associated with adult HSCs is involved in the transition of pre-HSC to embryonic HSCs. We propose an HSC developmental model in which pre-HSCs and embryonic HSCs sequentially give rise to adult types of HSCs in a stepwise manner.

## Introduction

Mammalian hematopoiesis develops in three distinct waves consisting of primitive hematopoiesis, definitive but transient hematopoiesis, and definitive and persistent hematopoiesis which is established by hematopoietic stem cells (HSCs) [1], [2]. Both the first and second hematopoietic waves originate from the yolk sac where hemangioblasts, common precursors of the hematopoietic and endothelial lineages likely play a crucial role [3]. However, whether HSCs arise in either the yolk sac or the paraaortic splanchnopleure/aorta-gonad-mesonephros (P-Sp/AGM) region remains controversial [4], [5], [6]. The relationship between HSCs and hemangioblasts is also obscure [2], [7]. In order to understand how HSCs develop in early embryos, it is important to determine the cellular origin of HSCs rather than the organ origin of HSCs.

Hematopoiesis and vasculogenesis in the early mouse embryo have been recapitulated well by *in vitro* ES differentiation systems [8], [9], [10]. However, generation of HSCs in substantial numbers from ESCs *in vitro* has been difficult. Kyba *et al.* were the first to report that HSCs can be efficiently generated from ESCs in the OP9 co-culture system by combining this with an inducible HOXB4 expression system (OP9 and iHOXB4 system) [11].

In concept, mesodermal cells first commit to the hematopoietic lineage before giving rise to HSCs. We provisionally called such cells pre-HSCs, and attempted to identify them in embryoid bodies (EB) using the OP9 and iHOXB4 system. We detected the potential to give rise to HSCs among c-Kit^+^CD41^+^CD45^−^ cells derived from ESCs on day 6 of culture (EB6). The presence of hematopoietic progenitor activity in this population has been described [12], [13], [14]. The present report, however, is the first to document the presence of pre-stem cell activity but little hemangioblast activity in the c-Kit^+^CD41^+^CD45^−^ cell population.

Pre-HSCs gave to an embryonic type of HSCs (embryonic HSCs) capable of reconstituting adult hematopoietic system but at a low degree. OP9 cells supported the transition of pre-HSCs to embryonic HSCs. Some genes were up- and down-regulated during the transition via enforced expression of HOXB4. Interestingly, about two-thirds of the markedly up-regulated genes were also found in our adult HSCs gene expression data. These results suggest that adult HSC-related molecules establish the very early stages of HSC development. Based on these results, we propose an HSC development model in which pre-HSCs through the stage of embryonic HSCs give rise to adult types of HSCs.

## Results

### Experimental design

**Figure 1:**
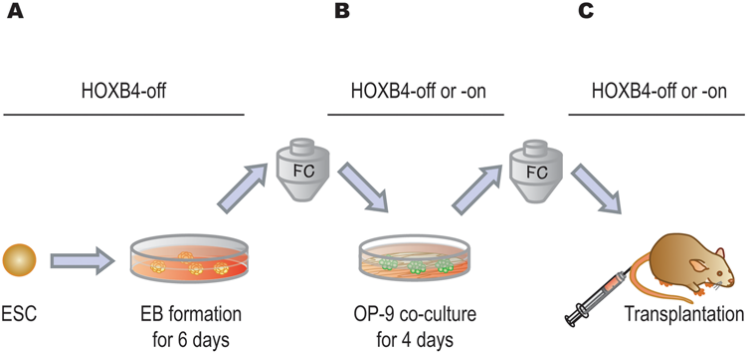
Study design. (A) iHOXB4 ESCs were differentiated into EBs for 6 days in the presence of Dox. (B) Dissociated EB cells were analyzed and sorted by flow cytometry (FC). EB cells or their subpopulations were co-cultured with OP9 cells for 4 days in the presence or absence of Dox. (C) Co-cultured cells were analyzed and GFP^+^ cells were sorted by FC when HOXB4 expression was turned on. Regardless of HOXB4 status in co-cultures, FC sorting was performed on co-cultured cells based on forward and side scatters and on surface markers. Sorted cells were subjected to long-term reconstitution assays. The sorting process turned out to be useful for cell counts, removal of dead cells, and elimination of the remaining Dox.

Our basic experimental strategy consisted of EB formation, co-culture with OP9 cells, and functional assays (Fig. 1). iHOXB4 ESCs were allowed to differentiate spontaneously into EBs for 6 days without HOXB4 expression. We decided to fractionate EB6 cells mainly because by day 6 of culture the number of multipotent progenitors reaches a plateau and several surface markers become detectable (Fig. S1). CD41 is known as a marker for the initiation of definitive hematopoiesis [15], [16], [17], [18], [19]. As shown in Fig. S1, CD41 appeared in a significant proportion of EB cells on day 6 of culture. Induced HOXB4 expression during EB formation did not affect the generation of colony forming cells and repopulating cells in the OP9 and iHOXB4 system or the appearance of surface markers in EB cells. EB6 cells were analyzed and sorted by flow cytometry. Sorted EB6 cells were co-cultured with OP9 cells for various days under HOXB4-on or -off conditions. The minimal requirement of the co-culture period appeared to be only 4 days (data not shown), which is much shorter than previously thought [11]. After a second analysis and fractionation by flow cytometry, cells were subjected to *in vivo* repopulating assays under HOXB4-on or -off conditions.

### The potential to give rise to HSCs in EB6 subpopulations

**Figure 2:**
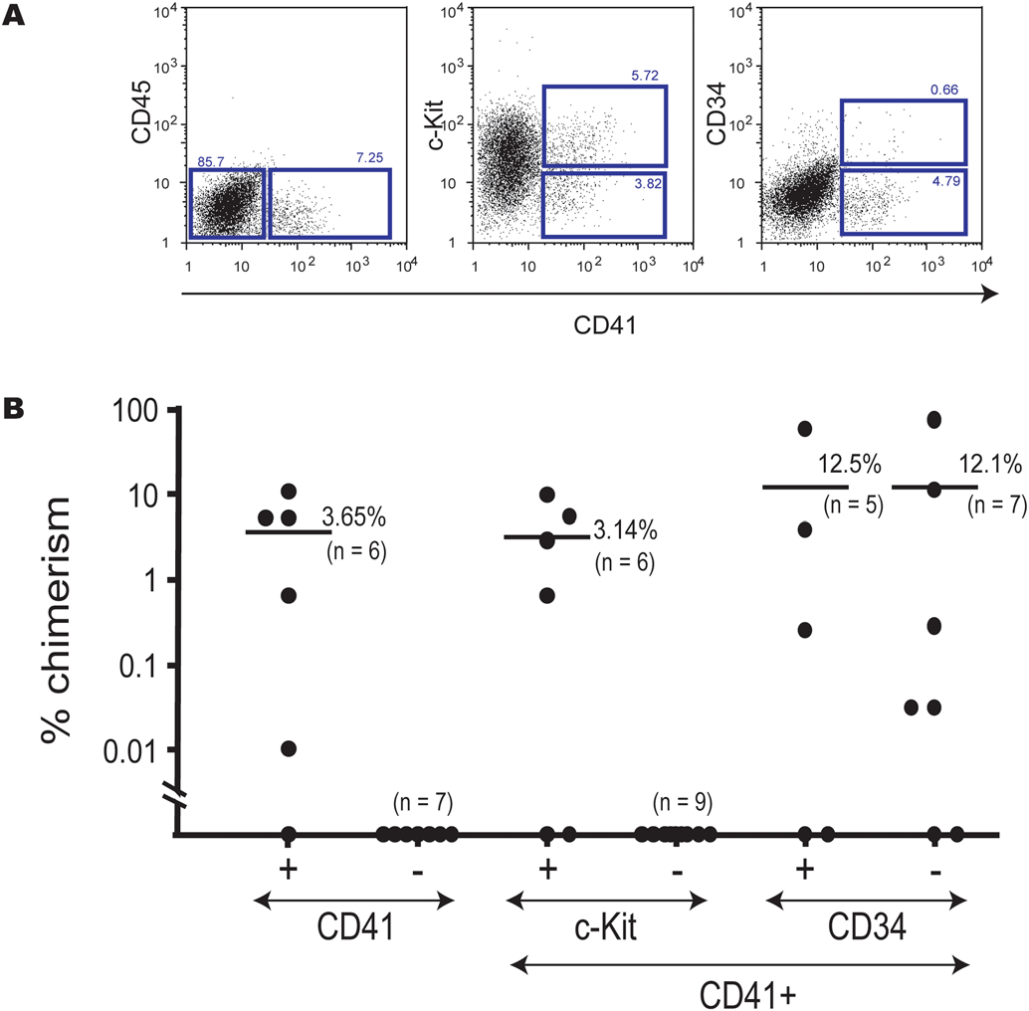
EB6 subpopulations with the potential of giving rise to HSCs. (A) Data from flow cytometry analysis show the expression of CD41, CD45, c-Kit, and CD34 in EB6 cells. The sorting gates for CD41^−^ or CD41^+^ cells, c-Kit^+^CD41^+^ or c-Kit^−^CD41^+^ cells, and CD34^+^ CD41^+^ or CD34^−^CD41^+^ cells are indicated as squares. (B) EB6 cells were fractionated based on expression of CD41, c-Kit, and CD34, co-cultured with OP9 cells for 4 days, sorted for GFP+ cells, and transplanted into lethally irradiated mice. HOXB4 expression was maintained from *in vitro* co-culture through *in vivo* repopulation. Recipient mice were analyzed 16 weeks after transplantation. Over 95% of reconstituted blood cells were of myeloid lineage in all cases (data not shown). Two independent experiments gave similar results. Data from one experiment are shown. See Table S1 for the number of transplanted cells from each subpopulation.

EB6 cells were stained with anti-CD41 antibody in combination with anti-CD45, -c-Kit, and -CD34 antibodies and others, and analyzed by flow cytometry (Fig. 2A). CD41^+^ and CD41^−^ cells, c-Kit^+^CD41^+^ and c-Kit^−^CD41^+^ cells, or CD34^+^CD41^+^ and CD34^−^CD41^+^ cells were sorted by flow cytometry. Sorted cells were co-cultured with OP9 cells for 4 days, and then transplanted into lethally irradiated mice along with rescue cells (Table S1) while HOXB4 expression was maintained from *in vitro* co-culture through *in vivo* repopulation.

Analysis of peripheral blood cells of the recipient mice 16 weeks after transplantation showed that c-Kit^+^CD41^+^ cells were enriched in cells with long-term repopulating activity (Fig. 2B). Long-term repopulating activity was similarly detected in both CD34^−^ and CD34^+^ cells. Lineage analysis of reconstituted mice showed that myeloid lineage reconstitution predominated. A very low level of B- and T-lymphoid lineage reconstitution was observed only in limited cases. As previously suggested, this might be due to an adverse effect of HOXB4 overexpression [11]. For long-term repopulation, HOXB4 needed to be expressed during the OP9 co-culture period and throughout the repopulation period (data not shown). All these data clearly show that c-Kit^+^CD41^+^ cells are the cells that require HOXB4 expression to manifest long-term repopulating activity. In addition, it should be emphasized that CD45 is not expressed in these cells (Fig. 2A).

### Hemangioblasts in EB6 subpopulations

**Figure 3:**
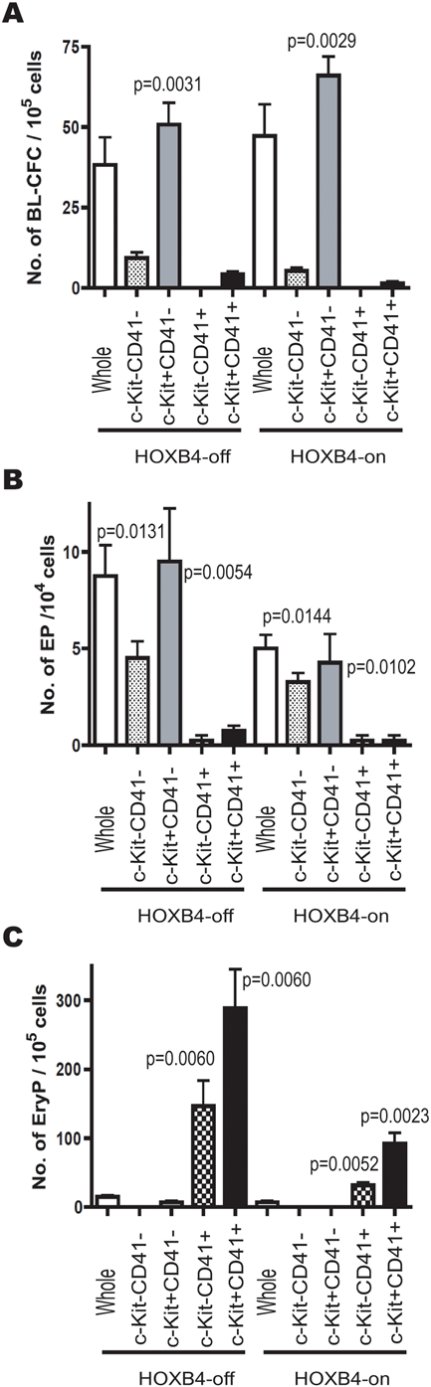
Hemangioblastic, endothelial, and primitive erythrocytic potentials among EB6 subpopulations. Colony forming abilities in unfractionated, c-Kit^−^CD41^−^, c-Kit^+^CD41^−^, c-Kit^−^CD41^+^, or c-Kit^+^CD41^+^ EB6 cells were examined in quadruplicate. (A) Blast colony-forming assays were performed. (B) Vascular endothelial progenitors (EP) were detected by the OP9 co-culture system with cytokines as previously described [20]. (C) Erythroid colonies were detected by methylcellulose colony assays. Detected erythroid colonies contained primitive erythrocytes as identified by *β-H1 globin* expression (data not shown). Kruskal-Wallis testing was performed for statistical analysis.

To examine whether c-Kit^+^CD41^+^ cells have hemangioblastic activity, we performed blast colony-forming cell (BL-CFC) assays [3] on EB6 cells under HOXB4-off or -on conditions. Unexpectedly, c-Kit^+^CD41^+^ cells exhibited scant BL-CFC activity, and c-Kit^+^CD41^−^ cells instead were significantly enriched in BL-CFC, regardless of HOXB4 expression status (Fig. 3A). The potentials to give rise to blood cells and vascular endothelial cells in BL-CFC were examined on a clonal basis as previously described [20]. Most of these blast colonies individually exhibited hematopoietic and/or endothelial differentiation potential (Fig. S2). Of note is that neither BL-CFCs nor cells composing blast colonies significantly respond to HOXB4 expression (Fig. 3A and Fig. S2). Consistent with BL-CFC data, most vasculogenic activity was detected in CD41^−^ cells (Fig. 3B). In contrast, most primitive erythropoietic activity was detected in CD41^+^ cells (Fig. 3C), as in the yolk sac (YS) [15], supporting the view that primitive hematopoietic progenitors arise soon after the development of mesoderm [21]. Primitive erythroid colony formation was significantly inhibited by HOXB4 expression (Fig 3C).

### Genes expressed in EB6 subpopulations

RT-PCR was performed on cDNAs prepared from fractionated EB6 cells. Consistent with recently published data[14], all genes examined, including *Runx1*, *Scl*, *Gata1*, and *Gata2*, were expressed in c-Kit^+^CD41^+^ cells (Fig. S3), suggesting that this population at this developmental stage is already in the process of establishing definitive hematopoiesis. Expression of *Gata1*, *β-H1 globin* (*Hbb-bh1*), and *β-major globin* (*Hbb-b1*), detected in c-Kit^−^CD41^+^ and c-Kit^+^CD41^+^ cells, supports observations that these two populations contain primitive erythrocyte precursors (EryPs). *Flk1* expression with faint *Brachyury* expression in both c-Kit^+^CD41^−^ cells and c-Kit^+^CD41^+^ cells implies that these populations are the immediate progeny of mesodermal precursors. Although low levels of endogenous mouse *HoxB4* expression were detected in c-Kit^+^CD41^+^ cells, higher expression levels might be required for generation of HSCs.

### Cell surface markers on long-term repopulating cells derived from EB cells

**Figure 4:**
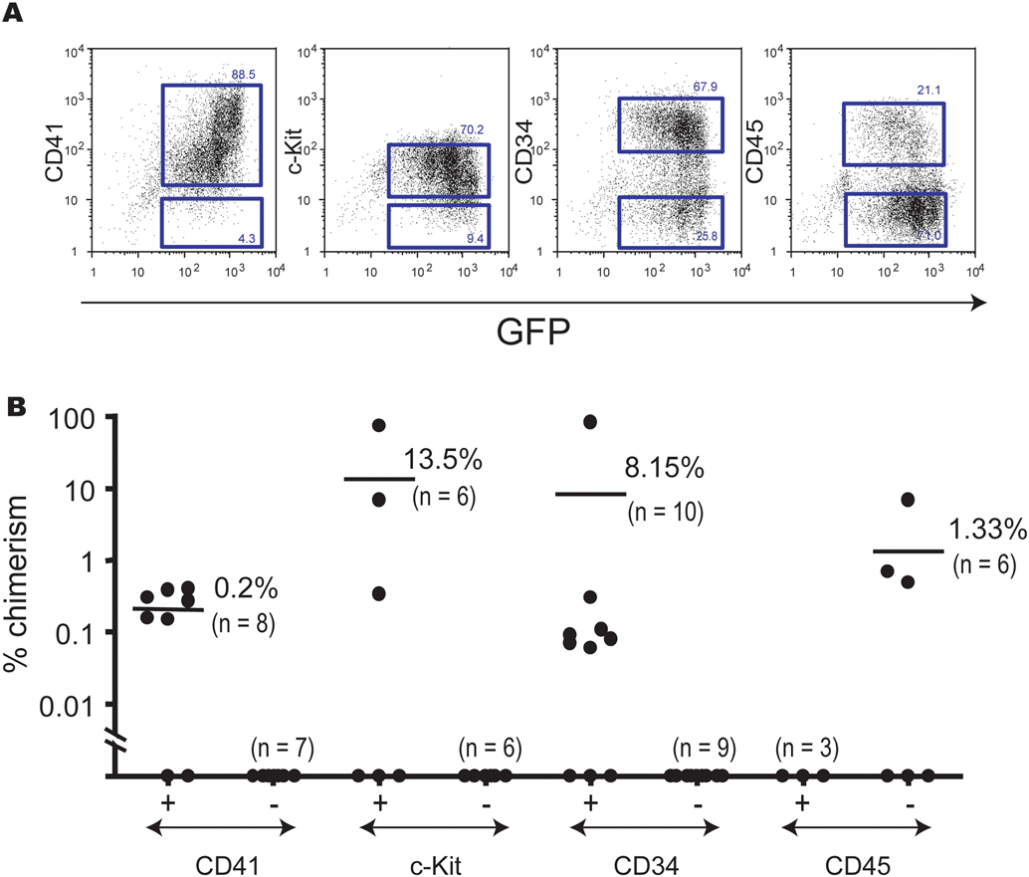
Surface markers of embryonic HSCs generated from EB6 cells *in vitro*. (A) CD41^+^ EB6 cells were co-cultured with OP9 cells in the absence of Dox for 4 days. Cells collected from the co-cultures were analyzed by flow cytometry. HOXB4-expressing cells were detected by GFP expression. Data show the expression of CD41, c-Kit, CD34, and CD45 in GFP^+^ cells. The sorting gates for CD41^−^ or CD41^+^ cells, c-Kit^−^ or c-Kit^+^ cells, CD34^−^ or CD34^+^ cells, and CD45^−^ or CD45^+^ cells are shown as squares. (B) Co-cultured cells were fractionated based on expression of CD41, c-Kit, CD34, and CD45, and were transplanted into lethally irradiated mice. Recipient mice were analyzed 16 weeks after transplantation. Over 95% of reconstituted blood cells were of myeloid lineage in all cases (data not shown). Two independent experiments gave similar results. Data from one experiment are shown. See Table S2 for the number of transplanted cells for each subpopulation.

In order to define HSC phenotypes just before transplantation, CD41^+^ EB6 cells were co-cultured with OP9 cells and were induced to express HOXB4. HOXB4-expressing cells, detected as GFP^+^ cells, were analyzed for expression of cell surface markers. GFP^+^ cells were fractionated based on expression of CD41, c-Kit, CD34, or CD45 by flow cytometry (Fig. 4A) and were transplanted into lethally irradiated mice with rescue cells (Table S2). Analysis of recipient mice 16 weeks after transplantation showed that CD41^+^ cells, c-Kit^+^ cells, CD34^+^ cells, and CD45^−^ cells were enriched in long-term repopulating activity (Fig. 4B). Myeloid lineage was predominantly reconstituted in all cases. Numbers of CD41^+^, c-Kit^+^, and CD34^+^ cells apparently decreased in the absence of HOXB4 expression (data not shown). These data indicate that HOXB4 expression selectively maintains the c-Kit^+^CD41^+^ CD45^−^ phenotype and up-regulates CD34 expression during the co-culture period. It is known that fetal liver HSCs express CD34 antigen while adult bone marrow HSCs barely express CD34 antigen [22], [23]. These data suggest that ESC-derived HSCs remain phenotypically immature.

### *In vivo* function of HSCs derived *in vitro* from ESCs

We observed that c-Kit^+^CD41^+^ cells had accumulated in the bone marrow of recipient mice when analyzed 18 weeks after transplantation (Fig. S4). We attempted to shut HOXB4 expression off in recipient mice from 8 to 14 weeks after transplantation by letting them drink water containing 100 µg/ml of Dox. Analysis of peripheral blood cells from these mice showed that GFP^+^ cells became undetectable and that B- and T-lymphoid lineage reconstitution was significantly improved. Myeloid lineage reconstitution, contrariwise, was reduced, with decreases in total chimerism (data not shown). After GFP^+^ bone marrow cells were isolated from other mice reconstituted with ESC-derived cells, 1.2×10^5^ GFP^+^ cells together with 2×10^5^ rescue cells was transplanted into each of 6 lethally irradiated mice, of which 3 were given Dox and 3 were not. Of the 3 recipients given Dox, 2 mice showed 0.8% and 15% chimerism, with respectively 46 ∶ 54% and 50 ∶ 50% myeloid ∶ lymphoid lineages. Of the 3 recipients not given Dox, 4 mice showed 45% and 83% chimerism with almost exclusively myeloid lineage. These results support the hypothesis that HOXB4 expression negatively affects lymphoid differentiation and positively affects repopulating activity in ES-derived HSCs [11], effects not seen in adult HSCs [24].

### HOXB4 target genes in HSC development

Our last experiments compared gene expression profiling among c-Kit^+^CD41^+^ EB6 cells (cells with the potential to give rise to HSCs), c-Kit^+^CD41^+^ cells after co-culture with HOXB4 expression (repopulating cells), and c-Kit^+^CD41^+^ cells after co-culture without HOXB4 expression (non-repopulating cells). We attempted to identify candidate genes whose expression is up- or down-regulated by HOXB4, among which might exist important genes that control the early development of HSCs. To verify the Tet-off strategy, HOXB4 expression was examined in these 3 populations and in ESCs maintained in the presence of Dox (ESCs without HOXB4 expression). As expected, *HOXB4* expression was only detected in c-Kit^+^CD41^+^ EB6 cells after co-culture without Dox (Fig. S5).

Microarray analysis was performed on cDNAs prepared from c-Kit^+^CD41^+^ EB6 cells without HOXB4 expression and c-Kit^+^CD41^+^ cells after co-culture with or without HOXB4 expression. In order to focus on genes up- and down-regulated by HOXB4 expression, we employed stringent criteria (Legends, Tables S3 and S4). Genes with 5-fold or more difference in gene chip scores between c-Kit^+^CD41^+^ cells after co-culture with HOXB4 expression and c-Kit^+^CD41^+^ cells without HOXB4 expression were selected.

**Figure 5:**
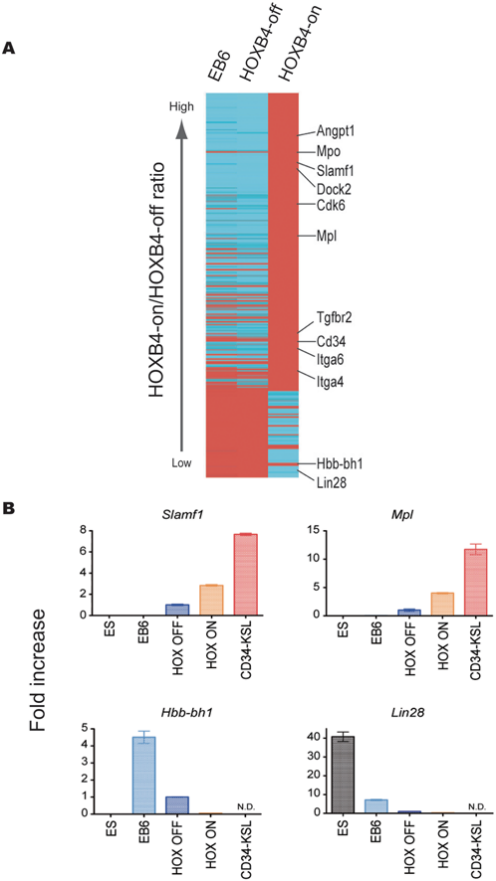
Heat map of differentially expressed genes. (A) Microarray analysis was performed on cDNAs from c-Kit^+^CD41^+^ EB6 cells without HOXB4 expression, c-Kit^+^CD41^+^ cells after co-culture without HOXB4 expression, and c-Kit^+^CD41^+^ cells after co-culture with HOXB4 expression. The finally selected 223 genes are shown in this graph (for selection criteria, see Legend, Table S3). Red indicates genes up-regulated 5-fold or more. Blue indicates genes down-regulated 5-fold or more. (B) Real-time PCR was performed on normalized cDNA from the cells described above, from ESCs maintained without HOXB4 expression, and from CD34^−^KSL cells from adult bone marrow. The mean plus or minus 1SD (n = 3) is shown for 4 representative genes.

After selection, 294 and 115 probes, respectively, remained for HOXB4 up- and down-regulated genes. We next examined whether these selected genes are expressed in HSCs via gene expression profiling of adult HSCs. HSC-expressing genes shared 200 of 294 probes for HOXB4 up-regulated genes. Of great interest is that *CD34*, *CD150* (*Slamf1*), *c-Mpl*, *integrin α4* and *α6* (*Itga4* and *6*), and *transforming growth factor β type II receptor* (*Tgfbr2*) were among them. On the other hand, 58 of 115 probes for down-regulated genes by HOXB4 were not detected on adult HSC profiling. All up-regulated adult HSC-related genes and all down-regulated adult HSC-unrelated genes are listed in Tables S3 and S4 and are also schematically presented as a heat map in Fig. 5A. The overall similarity in the heat map between the EB6 and the HOXB4-off samples suggests that these data represent the effect of enforced expression of HOXB4. RT-PCR analysis showed that the expression levels of *CD150* and *c-Mpl* were significantly higher in HOXB4-on c-Kit^+^CD41^+^ cells than in HOXB4-off c-Kit^+^CD41^+^ cells, and that the expression levels of *βh1-globin* (*Hbb-bh1*) and *Lin28* were significantly lower in HOXB4-on c-Kit^+^CD41^+^ cells than in HOXB4-off c-Kit^+^CD41^+^ cells (Fig. 5B). A marked increase in CD34^+^ repopulating cells (Fig. 4) and a marked decrease in primitive erythroid progenitors (Fig. 3) with HOXB4 expression were consistent with the gene expression profiling data.

A number of genes are implicated as playing important roles in the generation of HSCs. According to our microarray data, significant levels of Scl, Runx1, Gata2, or Lmo2, were expressed in c-Kit^+^CD41^+^ cells, but induced HOXB4 expression did not change their expression levels. Expression levels of Cdx1 and Cdx4 remained low in c-Kit^+^CD41^+^ cells regardless of HOXB4 expression.

## Discussion

This study demonstrates that pre-HSCs, perhaps conceptually relevant to hemogenic endothelium [25], can be prospectively isolated from developing mouse EBs. Pre-HSCs were unable to engraft and to reconstitute the hematopoietic system in lethally irradiated adult mice. To engraft in adult mice, pre-HSCs should acquire both engraftment and repopulating capacities. This developmental process was driven or enhanced by enforced expression of HOXB4. Contrary to previous studies [11], HOXB4 had to be continuously expressed *in vivo* after transplantation to maintain long-term repopulation in this study. When HOXB4 expression was turned off in some reconstituted mice, myeloid reconstitution level was decreased while B- and T-lymphoid reconstitution levels were increased. As a result, the total chimerism was gradually reduced (data not shown). We used a Tet-off system while Kyba et al. used a Tet-on system. An explanation for this discrepancy may be that Tet-on systems are “leaky” by comparison with Tet-off systems, permitting weak persistent expression of HOXB4 even after turn-off in the work of Kyba *et al*. Long-term repopulating cells generated from pre-HSCs by OP9 co-culture and HOXB4 expression persistently showed low levels of long-term reconstitution. When 10^5^ adult bone marrow cells, instead of Sca-1^−^ rescue cells, were used as competitor cells, reconstitution became undetectable (data not shown). A similar property has been observed for HSCs from the YS and P-Sp/AGM region. We operationally called these HSCs with low repopulating potential embryonic HSCs.

As previously noticed [11], HOXB4 overexpression seemed to have prevented lymphoid reconstitution, with long-term reconstitution mainly myeloid in this study. Multilineage reconstitution has been a criterion for HSCs. However, myeloid reconstitution may be more reliable than lymphoid reconstitution as a marker of HSC activity because short-lived granulocytes are never detectable in the circulation for long unless they are continuously supplied by engrafted HSCs.

Pre-HSCs and embryonic HSCs are distinct populations in function and gene expression profiling, but they both exhibited the c-Kit^+^CD41^+^CD45^−^ phenotype. Since c-Kit is already expressed in a significant proportion of undifferentiated ESCs and in most YS, P-Sp/AGM, fetal liver, and adult bone marrow HSCs [6], [26], [27], [28], the maintenance of this receptor tyrosine kinase may be crucial for the development of HSCs from the internal cell mass.

CD41 marks both primitive and definitive hematopoiesis [15], [16], [17], [18]. The developmental wave of definitive but transient hematopoiesis clearly differs from that of HSCs [1], [2]. Whether CD41 also marks HSCs in their early development has been uncertain. In this study, pre-HSCs and primitive erythroid progenitors were detected among CD41^+^ cells (Figs. 2B, 3C). In contrast, hemangioblastic and vasculogenic activities were principally detected in CD41^−^ cells.

Hemangioblasts are thought to play a major role in initiation of primitive and definitive hematopoiesis [3]. HSCs have been generally believed to arise from hemangioblasts [7]. Unlike previous studies[3], our blast colony assays were performed on EB6 cells instead of EB3.0 or 3.5 cells. This might be the reason that pre-HSC and hemangioblast activities were detected in the separated populations. Pre-HSCs may develop closely associated with hemangioblasts because these two types of cells arise from common mesodermal precursors at a very early point. It is important to clarify at which stage these cell classes separate from one another during development. Our data suggest that pre-HSCs are separated from hemangioblasts as soon as they arise. The possibility exists that pre-HSCs initially develop through hemangioblasts, but soon thereafter these two types of cells become distinct from one another. Alternatively, HSCs develop independent of hemangioblasts. Since *in vitro* differentiation of ESCs along the blood lineage mostly mimics YS hematopoiesis [29], it is possible that pre-HSCs arise in close association with YS development. In this case, pre-HSCs presumably are unable efficiently to differentiate into embryonic HSCs in the YS microenvironment, but, after migration, are able to do so in particular developmental niches like the P-Sp/AGM region [4], [5] and the fetal liver microenvironment.

Although fetal and adult HSCs express CD45, pre-HSCs and embryonic HSCs were shown not to express CD45. Most HSCs from the YS and P-Sp/AGM region at E10.5 or earlier do not express CD45 [19], [30]. In this regard, CD45 is a late maturation marker of HSCs whereas CD41 is an early maturation marker of HSCs. Identification and characterization of c-Kit^+^CD41^+^CD45^−^ pre-HSCs and embryonic HSCs in early developing embryos will clarify the significance of changes in HSC phenotype.

We and others have been interested in target genes of HOXB4. If other HSC inducers are indentified among such molecules, more efficient generation of HSCs should become possible. A large number of candidate target genes has been reported recently [31]. Unfortunately, these expressed genes were not always identified among populations properly enriched in HSC activity. We therefore used c-Kit^+^CD41^+^ cells from which HSC activity emerged after HOXB4 expression was turned on.

Among a number of candidate genes obtained in this study were many genes known to be expressed in adult HSCs (Table S3). Of special note is that *CD34*, *CD150*, and *c-Mpl* are up-regulated in the transition of pre-HSCs to embryonic HSCs. Only some pre-HSCs expressed CD34, but all embryonic HSCs derived from pre-HSCs expressed CD34 (Fig. 4). That CD34 is expressed in YS and P-Sp/AGM HSCs [6], [32] supports the inference that all these cells are closely related. CD150, which is expressed from fetal HSCs to adult HSCs, is a new HSC marker [33], [34]. c-Mpl, the receptor for TPO, is expressed in most fetal and adult HSCs. Although the function of CD34, CD150, or c-Mpl is not essential for the development of HSCs [12], [35], [36], it is suggested that these HSC markers begin to be expressed at the embryonic HSC stage. CD34, CD150, and c-Mpl could be good candidates for the earliest markers during HSC development. Since many intracellular molecules (*e.g.*, angiopoietin 1 and myeloperoxidase) were also up-regulated, they might also serve as markers for embryonic HSCs. Angiopoietin 1, secreted by HSCs in the P-Sp/AGM region, fetal liver, and adult bone marrow, has been suggested to promote angiogenesis [37]. High levels of expression of these molecules might be tightly associated with commitment to HSC lineage. The origin of HSCs – YS or P-Sp/AGM region – has been debated for a very long time. It is difficult to determine precisely which site is the first origin of HSCs because in the mouse embryo the P-Sp/AGM region does not exist at E7, when the YS begins to appear. A combination of markers listed in this study should be useful for *in vivo* detection of embryonic HSCs.

**Figure 6:**
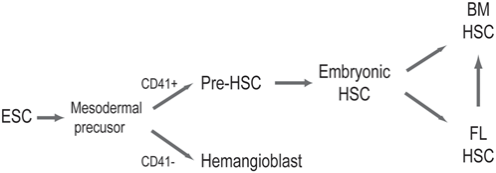
A stepwise developmental model for HSCs. We propose a working model for HSC development. Pre-HSCs originate from mesoderm, possibly independent of hemangioblasts; pre-HSC give rise to embryonic HSCs in particular niches in the YS or P-Sp/AGM region; embryonic HSCs give rise to fetal and adult HSCs in particular niches in the fetal liver and bone marrow.

Fetal and adult HSCs are functionally distinct [38], [39]. Pre-HSCs and embryonic HSCs are functionally different from fetal and adult HSCs. Our working model for HSC development is presented in Fig. 6. We propose that pre-HSCs which arise from mesoderm, possibly independent of hemangioblasts, give rise to embryonic HSCs which subsequently give rise to fetal and adult HSCs. Whether all adult HSCs are generated by fetal HSCs remains uncertain, as recently suggested [40]. These processes should take place in spatially and temporally established niches in developing embryos. Certainly much more work is required, but identification and characterization of pre-HSCs and embryonic HSCs in developing embryos are central to validation of this model.

Embryonic stem cells (ESCs) hold great promise to innovate a variety of new therapies for regenerative medicine because of their potential of differentiating into all sorts of adult cells. The key to success in stem cell therapy is to establish methods of properly differentiating ESCs into any particular type of tissue-specific stem cells. Recent establishment of induced pluripotent stem cell lines [41], [42] demands more such protocols. In order to generate HSCs from ESCs efficiently *in vitro*, optimal conditions must be determined for each developmental step in our model.

## Materials and Methods

### Mice

129/OlaHsd (129Ola) mice were purchased from Jackson Laboratory (Bar Harbor, ME). Ly5 congenic C57BL/6 mice (B6-Ly5.1) were obtained from Sankyo Laboratory Service (Tsukuba, Japan). 129Ola and B6-Ly5.1 mice were mated to produce F1 mice (Ly5.1×Ly5.2). Mice were maintained in the Institute of Medical Science University of Tokyo Animal Research Center. All experiments using mice received approval from the Institute of Medical Science Animal Experiment Committee.

### ESCs

The mouse ES cell line EB3, derived from E14tg2a ESCs, was maintained without mouse embryonic fibroblasts in Glasgow minimum essential medium supplemented with 10% fetal calf serum (FCS) (JRH Bioscience, Lenexa, KS), 0.1 mM 2-mercaptoethanol (2-ME), 2 mM L-glutamine (L-Gln), 0.1 mM non-essential amino acids, 1 mM sodium pyruvate (Invitrogen, Carlsbad, CA), 1,000 U/ml leukemia inhibitory factor (LIF, Chemicon, Temecula, CA), and 100 U/ml penicillin/streptomycin. For these cultures, a 100 mm-tissue culture dish was used after coating with 5 ml of 0.1% gelatin in PBS for 10 min at 37°C.

### Tet-regulated HOXB4/GFP expression in ESCs

The tetracycline (Tet)-off system was chosen because gene expression is more strictly controllable in the Tet-off system than in the Tet-on system [43]. ESCs carrying the Tet-off iHOXB4 expression cassette in the ROSA26 locus (iHOXB4 ESCs) were made as previously described (Fig. S6) [44].

### *In vitro* ESC differentiation

iHOXB4 ESCs were maintained in the presence of 1 µg/ml doxycycline (Dox), a tetracycline derivative. To allow ESCs to differentiate into EBs, ESCs were trypsinized and collected in complete EB differentiation medium (EBD) [3]. Cells were transferred into a 100-mm Petri dish at 2×10^5^ cells per 10 ml EBD. The medium was changed on day 4 of culture and every 2 days thereafter.

### Co-culture with OP9 cells

OP9 cells were maintained in α-MEM containing 15% FCS. 10^5^ OP9 cells were plated in each well of a 6-well tissue culture plate 2 days before starting co-culture. Developed EBs were treated with 0.25% trypsin for 4 min at 37°C and were disrupted to yield single cells. Co-cultures were employed with IMDM containing 20 ng/ml mouse stem cell factor (SCF) and 20 ng/ml human thrombopoietin (TPO), 10% FCS, 2 mM L-Gln, 0.1 mM 2-ME, and 100 U/ml penicillin/streptomycin. On specified days of co-culture, cells were recovered from the culture dishes for analysis and sorting on a flow cytometer.

### Flow cytometry analysis and sorting

ESCs, EB cells, and cells after OP9 co-culture were stained with phycoerythrin-conjugated (PE-) anti-Flk-1 (eBioscience, San Diego, CA), allophycocyanin-conjugated (APC-) anti-CD31, biotinylated anti-CD34, PE-anti-CD41, and APC-anti-c-Kit antibodies (BD Biosciences, San Jose, CA) on ice for 30 min. Streptavidin-APC-Cy7 (BD Biosciences) was used for detection of biotinylated antibody. Analysis and sorting were performed on a MoFlo (DAKO, Glostrup, Denmark).

### Methylcellulose colony assays

Cells were cultured in 1% methylcellulose containing 30% FCS, 1% bovine serum albumin, 2 mM L-glutamine, and 0.05 mM 2-ME. For colony assays, 10 ng/ml mouse interleukin-3, 10 ng/ml SCF, 2 U/ml human erythropoietin, and 50 ng/ml TPO were included. Cells were incubated at 37°C in a humidified atmosphere with 5% CO_2_ in air. Colonies were counted on day 10 of culture and individually picked up. Each colony was cytocentrifuged onto a glass slide for morphological examination with May-Gruenwald-Giemsa staining. Primitive erythroid colonies were counted on day 3 of culture. Blast colony assays were performed as previously described [16]. In brief, cells were cultured in IMDM containing 1% methylcellulose, 10% FCS, 4.5×10^−4^ M monothiolglycerol, 1% L-Gln, 25 µg/ml ascorbic acid, 300 µg/ml human saturated transferrin, 5 ng/ml vascular endothelial growth factor, 100 ng/ml SCF, and 5 ng/ml IL-6. Blast colonies were counted on day 4 of culture.

### Long-term reconstitution assays

2- to 3-month-old B6-Ly5.1×129Ola F1 mice (Ly5.1/Ly5.2) were irradiated at a dose of 900 cGy. ES-derived cells (Ly5.2) were transplanted into these mice (Ly5.1/ly5.2) along with 3×10^5^ Sca-1-depleted (Sca-1^−^) bone marrow cells from B6-Ly5.1×129Ola F1 mice as rescue cells. To prepare Sca-1^−^ cells, mononucleated cells were obtained by density gradient centrifugation using Ficoll-Paque PLUS (Amersham Biosciences, Uppsala, Sweden). Cells were stained with anti-Sca-1 antibody-conjugated magnetic beads (Miltenyi Biotec, Bergisch Gladbach, Germany). Sca-1^+^ cells were magnetically depleted using an LD column (Miltenyi Biotec).

Peripheral blood cells from recipient mice were analyzed 4 and 16 weeks after transplantation. After red blood cell lysis, cells were stained with biotinylated anti-CD45.1 antibody. After washing, cells were stained with PE-anti-CD4, PE-anti-CD8, APC-anti-Gr-1, APC-anti-Mac-1, and PE-Cy7-anti-B220 antibodies and with APC-Cy7-streptavidin. At least 10^5^ cells were analyzed, and data were collected on a FACS Aria (BD Biosciences). Test donor cells' contribution was detected with the GFP marker. Percentage chimerism was defined as percentage of GFP^+^ cells in peripheral leukocytes. Test donor cells were considered to have contained long-term repopulating cells when chimerism was over 0.01%.

### RT-PCR

PCR was performed on cDNAs from sorted cells as previously described [45]. Primers are listed in Table S5. The PCR program consisted of 38 cycles of 15 sec at 95°C, 15 sec at 56°C, and 20 sec at 72°C.

### Real-time PCR

The PCR primers were designed using a program provided by Roche (https://www.roche-applied-science.com/sis/rtpcr/upl/index.jsp). PCR contained normalized cDNAs, Universal Probe Library Set, and FastStart Universal Probe Master (Roche, Basel, Switzerland). Quantitative PCR analyses were performed in real-time using an ABI PRISM 7900HT (Applied Biosystems, Foster City, CA). The PCR program consisted of 43 cycles of 15 sec at 95°C and 60 sec at 60°C. Each value was divided by the mean value from HOXB4-off samples to be expressed as fold increase.

### Microarray analysis

Total RNA was extracted from 3 sets of cells. The first was c-Kit^+^CD41^+^ EB6 cells derived from iHOXB4 ESCs without HOXB4 expression. The second and third were c-Kit^+^CD41^+^ cells after co-culture with OP9 cells for 4 days with or without HOXB4 expression. To prepare cells in the last 2 groups, c-Kit^+^CD41^+^ EB6 cells were plated onto a monolayer of OP9 cells and were cultured either in the presence or absence of Dox for 4 days. c-Kit^+^CD41^+^ cells were again separated from the co-cultures by flow cytometry. To compare gene expression profiles with those of adult HSCs, CD34^−^c-Kit^+^Sca-1^+^Lineager marker^−^ (CD34^−^KSL) cells were isolated from C57BL/6 mice as previously described [26]. In order to analyze cycling adult HSCs, CD34^−^KSL cells were incubated with 50 ng/ml SCF and 50 ng/ml TPO for 24 hours [46]. Integrity of RNA was assessed qualitatively on an Agilent 2100 Bioanalyzer (Agilent Technologies, Santa Clara, CA). cDNA was synthesized with a MessageAmp aRNA Kit (Applied Biosystems). *In vitro* transcription and labeling were performed using One-Cycle Target Labeling and Control Reagents (Affymetrix, Santa Clara, CA) for ESCs and ESC-derived cells, and using Tow-Cycle Target Labeling and Control Reagents (Affymetrix) for adult HSCs. The heat-fragmented probes were hybridized to a Mouse Genome 430 2.0 GeneChip (Affymetrix). The arrays were scanned and analyzed with the Affymetrix GeneChip System. The relative abundance of each gene was estimated from the average difference of intensities.

### Statistical analysis

Mann-Whitney testing was performed when two groups were compared. Kruskal-Wallis testing was performed when multiple groups were compared.

## Supporting Information

Table S1 (0.02 MB PDF)

Table S2 (0.02 MB PDF)

Table S3 (0.18 MB PDF)

Table S4 (0.06 MB PDF)

Table S5 (0.03 MB PDF)

Figure S1 Effect of HOXB4 expression in generation of CFU-nmEM and surface marker expression. (A) Data show colony formation by 1,000 cells derived from EB. 1,000 cells were obtained from EBs on days 0, 2, 4, 5, 6, and 7 of culture and co-cultured with OP9 cells for 4 days. Cells collected from the co-cultures were plated in methylcellulose. HOXB4 expression was turned off in both OP9 co-cultures and methylcellulose cultures (HOXB4-off), turned on in both (HOXB4-on), or turned on only in OP9 co-cultures (HOXB4-on to -off). The number of colonies was counted and cells composing each colony were morphologically examined. The graphs show the numbers of neutrophil/macrophage/erythroblasts/megakaryocyte (nmEM) colonies extracted from various colonies formed. Continuous expression of HOXB4 in methylcellulose culture was not necessary for nmEM colony formation. The number of colonies is the mean from 2 independent experiments. (B) Cell surface markers were examined during EB formation without HOXB4 expression. Flk-1+ cells were first detected on day 3 of culture; the proportion of Flk-1+ cells markedly increased on the following day and decreased thereafter. CD31+ cells and CD41+ cells became detectable by day 5 and day 6, respectively, followed by the appearance of CD34+ cells. CD45+ cells became detectable after day 6, but remained low in number. c-Kit+ cells constituted about half of the cells throughout EB formation regardless of whether HOXB4 expression was induced (data not shown).
(9.39 MB TIF)

Figure S2 Differentiation potential of individual blast colonies. Both hematopoietic and endothelial potentials were examined for individual blast colonies. Blast colonies were formed by whole EB6 cells (Fig. 3A). Colonies were individually picked up from methylcellulose and co-cultured with OP9 cells in the presence of vascular endothelial growth factor, stem cell factor, interleukin-3, TPO, and erythropoietin for 7 days. (A) The summary of hematopoietic and endothelial potentials detected in individual blast colonies. (B) Representative photomicrographs show that a colony consisted of blood cells and CD31-positive vascular endothelial cells.
(6.61 MB TIF)

Figure S3 RT-PCR for 4 EB6 cell populations. PCR was performed on cDNAs prepared from fractionated EB6 cells.
(3.55 MB TIF)

Figure S4 Analysis of bone marrow cells from recipient mice of HOXB4-expressing ES-derived cells. c-Kit+CD41+ EB6 cells were co-cultured with OP9 cells while HOXB4 was enforcedly expressed. After co-culture with OP9 cells, GFP+ cells and rescue cells were transplanted into lethally irradiated mice. 18 weeks after transplantation, bone marrow cells of the recipient mice were stained with antibodies and analyzed on a flow cytometer. (A–E) GFP− cells were derived from rescue cells and possibly from host cells. (F–J) GFP+ cells were derived from ESCs. GFP− cells and GFP+ cells are separately displayed for the expression of Gr-1 and Mac-1 (A, F), B220 and CD19 (B, G), CD4 and CD8 (C, H), Sca-1 and c-Kit (D, I), and CD41 and CD45 (E, J).
(1.28 MB TIF)

Figure S5 RT-PCR analysis for induced HOXB4 expression in c-Kit+CD41+ cells. ESCs were maintained in the presence of Dox (ES HOXB4-off). After ESCs were differentiated into EB6 cells in the presence of Dox, c-Kit+CD41+ cells were isolated (c-Kit+CD41+ EB6 HOXB4-off). These cells were co-cultured with OP9 cells in the presence or absence of Dox for 4 days, followed by recovery of c-Kit+CD41+ cells from the co-cultures (c-Kit+CD41+ HOXB4-off and c-Kit+CD41+ HOXB4-on). These c-Kit+CD41+ cells and ESCs along with adult bone marrow cells (Total BM) as a negative control were examined for HOXB4 expression by RT-PCR analysis. The PCR program consisted of 15 sec at 95°C, 15 sec at 60°C, and 30 sec at 72°C. A total of 42 cycles or 30 cycles was used for amplification of HOXB4 or Gapdh.
(9.28 MB TIF)

Figure S6 Tet-off inducible HOXB4/EGFP expression system. (A) Schematic presentation of the Tet-off HOXB4 gene expression cassette integrated into the constitutive active ROSA 26 locus on chromosome 6. HOXB4 cDNA was kindly provided by Dr. K. Humphries (Terry Fox Laboratory, Vancouver, Canada). The Tet-off regulated gene expression plasmid comprised a splice-acceptor (SA) sequence; a loxP-flanked neomycin phosphotransferase gene (neor) gene, including a polyA signal; the Tet-controlled transcriptional activator (tTA) gene, including a polyA signal; an insulator sequence; the tTA-responsive element (TRE), followed by the minimal immediate-early promoter from Cytomegalovirus (CMV); the rabbit beta-globin 2nd intron; human HOXB4 cDNA; an internal ribosome entry site (ires); EGFP cDNA; and a polyA signal. The constructed vector was amplified in E. coli Stabl2 cells (Invitrogen), purified using a GENOPURE plasmid maxi kit (Roche), linearized by SwaI digestion, and used to transfect ESCs. The Tet-regulated HOXB4/EGFP expression cassette was integrated into the constitutively active ROSA26 locus in EB3 cells by homologous recombination. In brief, EB3 ESCs were electroporated with the linearized vector and were selected with G418 (150–200 µg/ml). G418-resistant colonies were picked and ES clones carrying a targeted integration of the vector in the ROSA26 locus were identified by long distance-PCR analysis using the following primers: forward (ROSA26 locus 1st exon), 5′-CCTCGGCTAGGTAGGGGATCGGGACTCT-3′; reverse (neor gene), 5′-CGGAGAACCTGCGTGCAATCCATCTTGTTC-3′; forward (EGFP), 5′-GGATCACTCTCGGCATGGACGAGCTGTAC-3′; and reverse (ROSA26 locus 2nd exon), 5′-AGCCTTAAACAAGCACTGTCCTGTCCTCAAG-3′. The PCR cycles consisted of one cycle at 94°C for 1 min, 32 cycles at 98°C for 20 s, 66°C for 30 s, 68°C for 4 min, and one cycle at 72°C for 10 min. To remove the loxP-flanked neor gene, Cre recombinase was transiently expressed in the selected clones by transfection with the pCAG-cre-IRES-puro plasmid. The resultant ES cell line was named “inducible HOXB4-EGFP ESCs” (iHOXB4 ESCs). (B) In the absence of doxycycline (Dox), tTA binds to the TRE, resulting in activation of HOXB4/EGFP transcription. In the presence of Dox, Dox binds tTA, preventing tTA binding to the TRE. We tested if this inducible expression system works in HOXB4 ES clones. Representative results from Western blot analysis for 4 clones are shown. HOXB4 was not detected when ESCs were cultured in the presence of Dox. HOXB4 was detected when ESCs were cultured in the absence of Dox. Anti-FLAG antibody was used to detect HOXB4. EGFP expression in these ES clones was consistent with results from Western blots (data not shown).
(1.10 MB TIF)

## References

[1] Moore MAS, Lanza R, Gearhart J, Hogan B, Melton D, Pedersen R (2004). Ontogeny of the hematopoietic system.. Handbook of stem cells.

[2] Jaffredo T, Nottingham W, Liddiard K, Bollerot K, Pouget C (2005). From hemangioblast to hematopoietic stem cell: an endothelial connection?. Exp Hematol.

[3] Kennedy M, Firpo M, Choi K, Wall C, Robertson S (1997). A common precursor for primitive erythropoiesis and definitive haematopoiesis.. Nature.

[4] Muller AM, Medvinsky A, Strouboulis J, Grosveld F, Dzierzak E (1994). Development of hematopoietic stem cell activity in the mouse embryo.. Immunity.

[5] Cumano A, Ferraz JC, Klaine M, Di Santo JP, Godin I (2001). Intraembryonic, but not yolk sac hematopoietic precursors, isolated before circulation, provide long-term multilineage reconstitution.. Immunity.

[6] Yoder MC, Hiatt K, Dutt P, Mukherjee P, Bodine DM (1997). Characterization of definitive lymphohematopoietic stem cells in the day 9 murine yolk sac.. Immunity.

[7] Lacaud G, Robertson S, Palis J, Kennedy M, Keller G (2001). Regulation of hemangioblast development.. Ann N Y Acad Sci.

[8] Keller G, Kennedy M, Papayannopoulou T, Wiles MV (1993). Hematopoietic commitment during embryonic stem cell differentiation in culture.. Mol Cell Biol.

[9] Choi K, Kennedy M, Kazarov A, Papadimitriou JC, Keller G (1998). A common precursor for hematopoietic and endothelial cells.. Development.

[10] Nishikawa SI, Nishikawa S, Hirashima M, Matsuyoshi N, Kodama H (1998). Progressive lineage analysis by cell sorting and culture identifies FLK1+VE-cadherin+ cells at a diverging point of endothelial and hemopoietic lineages.. Development.

[11] Kyba M, Perlingeiro RC, Daley GQ (2002). HoxB4 confers definitive lymphoid-myeloid engraftment potential on embryonic stem cell and yolk sac hematopoietic progenitors.. Cell.

[12] Wang N, Satoskar A, Faubion W, Howie D, Okamoto S (2004). The cell surface receptor SLAM controls T cell and macrophage functions.. J Exp Med.

[13] Lengerke C, Schmitt S, Bowman TV, Jang IH, Maouche-Chretien L (2008). BMP and Wnt specify hematopoietic fate by activation of the Cdx-Hox pathway.. Cell Stem Cell.

[14] McKinney-Freeman SL, Lengerke C, Jang IH, Schmitt S, Wang Y (2008). Modulation of murine embryonic stem cell-derived CD41+c-kit+ hematopoietic progenitors by ectopic expression of Cdx genes.. Blood.

[15] Ferkowicz MJ, Starr M, Xie X, Li W, Johnson SA (2003). CD41 expression defines the onset of primitive and definitive hematopoiesis in the murine embryo.. Development.

[16] Mikkola HK, Fujiwara Y, Schlaeger TM, Traver D, Orkin SH (2003). Expression of CD41 marks the initiation of definitive hematopoiesis in the mouse embryo.. Blood.

[17] Emambokus NR, Frampton J (2003). The glycoprotein IIb molecule is expressed on early murine hematopoietic progenitors and regulates their numbers in sites of hematopoiesis.. Immunity.

[18] Bertrand JY, Giroux S, Golub R, Klaine M, Jalil A (2005). Characterization of purified intraembryonic hematopoietic stem cells as a tool to define their site of origin.. Proc Natl Acad Sci U S A.

[19] Matsubara A, Iwama A, Yamazaki S, Furuta C, Hirasawa R (2005). Endomucin, a CD34-like sialomucin, marks hematopoietic stem cells throughout development.. J Exp Med.

[20] Furuta C, Ema H, Takayanagi S, Ogaeri T, Okamura D (2006). Discordant developmental waves of angioblasts and hemangioblasts in the early gastrulating mouse embryo.. Development.

[21] Fujimoto T, Ogawa M, Minegishi N, Yoshida H, Yokomizo T (2001). Step-wise divergence of primitive and definitive haematopoietic and endothelial cell lineages during embryonic stem cell differentiation.. Genes Cells.

[22] Ito T, Tajima F, Ogawa M (2000). Developmental changes of CD34 expression by murine hematopoietic stem cells.. Exp Hematol.

[23] Matsuoka S, Ebihara Y, Xu M, Ishii T, Sugiyama D (2001). CD34 expression on long-term repopulating hematopoietic stem cells changes during developmental stages.. Blood.

[24] Sauvageau G, Thorsteinsdottir U, Eaves CJ, Lawrence HJ, Largman C (1995). Overexpression of HOXB4 in hematopoietic cells causes the selective expansion of more primitive populations in vitro and in vivo.. Genes Dev.

[25] Smith RA, Glomski CA (1982). “Hemogenic endothelium” of the embryonic aorta: Does it exist?. Dev Comp Immunol.

[26] Osawa M, Hanada K, Hamada H, Nakauchi H (1996). Long-term lymphohematopoietic reconstitution by a single CD34-low/negative hematopoietic stem cell.. Science.

[27] Morrison SJ, Hemmati HD, Wandycz AM, Weissman IL (1995). The purification and characterization of fetal liver hematopoietic stem cells.. Proc Natl Acad Sci U S A.

[28] Okada S, Nakauchi H, Nagayoshi K, Nishikawa S, Nishikawa S (1991). Enrichment and characterization of murine hematopoietic stem cells that express c-kit molecule.. Blood.

[29] Keller G (2005). Embryonic stem cell differentiation: emergence of a new era in biology and medicine.. Genes Dev.

[30] Nobuhisa I, Ohtsu N, Okada S, Nakagata N, Taga T (2007). Identification of a population of cells with hematopoietic stem cell properties in mouse aorta-gonad-mesonephros cultures.. Exp Cell Res.

[31] Schiedlmeier B, Santos AC, Ribeiro A, Moncaut N, Lesinski D (2007). HOXB4's road map to stem cell expansion.. Proc Natl Acad Sci U S A.

[32] Sanchez MJ, Holmes A, Miles C, Dzierzak E (1996). Characterization of the first definitive hematopoietic stem cells in the AGM and liver of the mouse embryo.. Immunity.

[33] Kiel MJ, Yilmaz OH, Iwashita T, Yilmaz OH, Terhorst C (2005). SLAM family receptors distinguish hematopoietic stem and progenitor cells and reveal endothelial niches for stem cells.. Cell.

[34] Kim I, He S, Yilmaz OH, Kiel MJ, Morrison SJ (2006). Enhanced purification of fetal liver hematopoietic stem cells using SLAM family receptors.. Blood.

[35] Suzuki A, Andrew DP, Gonzalo JA, Fukumoto M, Spellberg J (1996). CD34-deficient mice have reduced eosinophil accumulation after allergen exposure and show a novel crossreactive 90-kD protein.. Blood.

[36] Abkowitz JL, Chen J (2007). Studies of c-Mpl function distinguish the replication of hematopoietic stem cells from the expansion of differentiating clones.. Blood.

[37] Takakura N, Watanabe T, Suenobu S, Yamada Y, Noda T (2000). A role for hematopoietic stem cells in promoting angiogenesis.. Cell.

[38] Bowie MB, McKnight KD, Kent DG, McCaffrey L, Hoodless PA (2006). Hematopoietic stem cells proliferate until after birth and show a reversible phase-specific engraftment defect.. J Clin Invest.

[39] Kim I, Saunders TL, Morrison SJ (2007). Sox17 dependence distinguishes the transcriptional regulation of fetal from adult hematopoietic stem cells.. Cell.

[40] Samokhvalov IM, Samokhvalova NI, Nishikawa S (2007). Cell tracing shows the contribution of the yolk sac to adult haematopoiesis.. Nature.

[41] Takahashi K, Tanabe K, Ohnuki M, Narita M, Ichisaka T (2007). Induction of pluripotent stem cells from adult human fibroblasts by defined factors.. Cell.

[42] Yu J, Vodyanik MA, Smuga-Otto K, Antosiewicz-Bourget J, Frane JL (2007). Induced pluripotent stem cell lines derived from human somatic cells.. Science.

[43] Mizoguchi H, Hayakawa T (2002). The tet-off system is more effective than the tet-on system for regulating transgene expression in a single adenovirus vector.. J Gene Med.

[44] Miyazaki S, Miyazaki T, Tashiro F, Yamato E, Miyazaki J (2005). Development of a single-cassette system for spatiotemporal gene regulation in mice.. Biochem Biophys Res Commun.

[45] Zhou S, Schuetz JD, Bunting KD, Colapietro AM, Sampath J (2001). The ABC transporter Bcrp1/ABCG2 is expressed in a wide variety of stem cells and is a molecular determinant of the side-population phenotype.. Nat Med.

[46] Ema H, Takano H, Sudo K, Nakauchi H (2000). In vitro self-renewal division of hematopoietic stem cells.. J Exp Med.

