# Supplementary material for: Stepwise Development of Hematopoietic Stem Cells from Embryonic Stem Cells
Source: PLoS One. 2009 Mar 16;4(3):e4820. doi: 10.1371/journal.pone.0004820 (PMC2653650; doi:10.1371/journal.pone.0004820)
Supplement: Table S1 — (0.02 MB PDF) [file pone.0004820.s001.pdf]

Table S1 Numbers of EB6 cells sorted for co-culture and of co-cultured cells sorted for transplantation

Subpopulations of EB6 cells	% cells on flow cytometry	No. of sorted cells for co-culture	No. of sorted cells for transplantation
$CD41^+$	7.2	$1.9x10^6$	$1.0x10^7$
$CD41^-$	92	$2.0x10^7$	$2.0x10^6$
$c\text{-}Kit^+CD41^+$	5.3	$1.4x10^6$	$1.5x10^7$
$c\text{-}Kit^-CD41^+$	1.7	$5.0x10^6$	$6.0x10^5$
$CD34^+CD41^+$	6.5	$2.0x10^6$	$2.5x10^6$
$CD34^-CD41^+$	0.5	$1.1x10^5$	$1.2x10^7$

$CD41^+$ and $CD41^-$ cells, $c\text{-}Kit^+CD41^+$ and $c\text{-}Kit^-CD41^+$ cells, or $CD34^+CD41^+$ and $CD34^-CD41^+$ cells were simultaneously sorted by flow cytometry. Sorted cells were co-cultured with OP9 cells for 4 days under the HOXB4-on condition. GFP^+ cells were separated from the co-cultures by flow cytometry. For each group of cell populations, one tenth of the volume of buffer containing GFP^+ cells was transplanted into each of 5-10 lethally irradiated mice.
