# Supplementary material for: Stepwise Development of Hematopoietic Stem Cells from Embryonic Stem Cells
Source: PLoS One. 2009 Mar 16;4(3):e4820. doi: 10.1371/journal.pone.0004820 (PMC2653650; doi:10.1371/journal.pone.0004820)
Supplement: Table S2 — (0.02 MB PDF) [file pone.0004820.s002.pdf]

Supplemental Table S2 Numbers of co-cultured cells for transplantation

Subpopulations of co-cultured cells	% cells on flow cytometry	No. of cells sorted for transplantation
$CD41^{+}$	82	5.5×10^{6}
$CD41^{-}$	2	1.1×10^{5}
$c\text{-}Kit^{+}$	60	4.0×10^{6}
$c\text{-}Kit^{-}$	15	1.0×10^{6}
$CD34^{+}$	55	4.5×10^{6}
$CD34^{-}$	15	1.0×10^{6}
$CD45^{+}$	20	1.3×10^{6}
$CD45^{-}$	60	4.0×10^{6}

1.5×10^{6} $CD41^{+}$ EB6 cells were co-cultured with OP9 cells for 4 days. $CD41^{+}$ and $CD41^{-}$ cells, $c\text{-}Kit^{+}$ and $c\text{-}Kit^{-}$ cells, $CD34^{+}$ and $CD34^{-}$ cells, or $CD45^{+}$ and $CD45^{-}$ cells were simultaneously sorted from the GFP^{+} fraction by flow cytometry. For each group of cell populations, one tenth of the volume of buffer containing GFP^{+} cells was transplanted into each of 5-10 lethally irradiated mice.
