# Supplementary material for: Stepwise Development of Hematopoietic Stem Cells from Embryonic Stem Cells
Source: PLoS One. 2009 Mar 16;4(3):e4820. doi: 10.1371/journal.pone.0004820 (PMC2653650; doi:10.1371/journal.pone.0004820)
Supplement: Table S3 — (0.18 MB PDF) [file pone.0004820.s003.pdf]

Table S3 Adult HSC-related genes up-regulated by HOXB4

Gene	Gene Title	Genbank	Map	MOE430v2	EB6	HOXB4-off	HOXB4-on	Hibernating HSC	Cycling HSCs
Samd9l	sterile alpha motif domain containing 9-like	BB145092	6 A1-A2	1460603_at	7.600	0.300	347.000	632.300	516.600
Serpina3g	serine (or cysteine) peptidase inhibitor, clade A, member 3G	BC002065	12 E	1424923_at	12.600	2.400	2103.400	4621.100	4724.700
Arhgap15	Rho GTPase activating protein 15	BM246535	2 B	1435959_at	4.700	0.900	330.500	551.000	184.100
Prg1	proteoglycan 1, secretory granule	NM_011157	10 B4	1417426_at	27.300	13.200	3970.301	4937.600	2930.400
Cd69	CD69 antigen	AK017979	6 F3	1428735_at	4.100	1.300	347.500	210.100	82.000
Ptprc	protein tyrosine phosphatase, receptor type, C	NM_011210	1 E4	1422124_a_at	1.500	3.900	982.700	2751.100	1439.200
Rgs18	regulator of G-protein signaling 18	BB139986	1 F	1420398_at	14.200	4.000	927.300	849.500	977.200
			1 F	1449856_at	8.100	5.400	508.400	931.300	1014.800
Arhgap30	Rho GTPase activating protein 30	BM244999	1 H3	1436171_at	17.900	2.200	490.700	202.300	146.500
Slco4a1	solute carrier organic anion transporter family, member	AV348121	2 H4	1438160_x_at	14.600	1.300	286.100	507.500	2869.100
Gp49a	glycoprotein 49 A	U05264	10 B3 ; 10 B	1420394_s_at	37.300	11.200	2348.600	22.700	222.900
Pscdbp	pleckstrin homology, Sec7 and coiled-coil domains, binding	BC007144	2 C1.1	1451206_s_at	0.900	1.600	325.600	1039.000	520.500
		BB503614	2 C1.1	1435697_a_at	4.400	15.900	479.500	2457.500	1222.200
Coro1a	coronin, actin binding protein 1A	BB740218	7 F3	1455269_a_at	20.400	7.400	1190.600	1695.400	2022.200
Arhgef6	Rac/Cdc42 guanine nucleotide exchange factor (GEF) 6	BM246754	X A5	1429012_at	7.600	2.300	365.500	965.700	745.600
Ifi203	interferon activated gene 203	M74124	1 H3	1426906_at	1.300	2.200	345.200	1827.200	1088.400
			1 H3	1452231_x_at	3.200	6.700	387.000	1881.800	919.100
Cd53	CD53 antigen	NM_007651	3 F2.3	1448617_at	18.100	5.900	878.200	103.900	581.800
Psmb8	proteosome (prosome, macropain) subunit, beta type	NM_010724	17 B1	1422962_a_at	7.500	2.600	354.500	760.600	1180.300
Coro1a	coronin, actin binding protein 1A	BC002136	7 F3	1416246_a_at	30.300	15.600	2085.800	1620.800	2927.100
Glipr1	GLI pathogenesis-related 1 (glioma)	BC025083	10 D1	1424927_at	20.300	8.500	968.400	996.500	2757.400
Evi2b	ecotropic viral integration site 2b	AI122415	11 B5	1426505_at	15.500	2.600	295.800	540.900	296.000
Anxa1	annexin A1	NM_010730	19 B	1448213_at	60.800	2.500	273.600	2153.600	1017.800
D12Ertd647e	DNA segment, Chr 12, ERATO Doi 647, expressed	AW554405	12 E	1454757_s_at	3.100	3.500	362.000	636.000	582.900
		BI655075	12 E	1452956_a_at	24.300	26.200	493.700	373.600	511.800
Rtp4	receptor transporter protein 4	BC024872	16 B1	1418580_at	7.000	5.600	481.900	351.400	1462.600
Bcl11a	B-cell CLL	BB424718	11 A3.2	1456632_at	10.100	2.800	203.700	293.400	199.900
2410127E16Rik	RIKEN cDNA 2410127E16 gene	BF731393	11 A3.2	1457072_at	9.200	5.600	246.200	213.700	140.200
Plac8	placenta-specific 8	AF263458	5 E3	1451335_at	65.500	24.900	1781.100	388.700	255.700
Ltb	lymphotoxin B	NM_008518	17 B1	1419135_at	13.100	4.100	273.900	1064.000	382.800
Cxcl4	chemokine (C-X-C motif) ligand 4	NM_019932	5 E1	1448995_at	43.000	54.400	3525.502	655.900	449.700
Prtn3	proteinase 3	U97073	10 C1	1419669_at	13.100	9.700	612.600	1781.900	275.000
Gpr171	G protein-coupled receptor 171	BB229616	3 D	1438439_at	0.700	4.200	255.000	539.800	337.900
Phb	prohibitin	BM243379		1435144_at	9.900	7.300	436.300	201.400	316.000
Rac2	RAS-related C3 botulinum substrate 2	NM_009008	15 E1	1417620_at	36.400	18.100	1071.200	826.000	851.600
Angpt1	angiopoietin 1	BB453314	15 B3.1	1439066_at	35.000	13.500	706.700	2552.500	1058.200
Lzp-s	P lysozyme structural	AV066625	10 D2	1436996_x_at	19.300	16.700	829.700	3204.800	976.100
		AV058500	10 D2	1439426_x_at	11.200	17.300	429.600	2728.200	218.400
Arhgap9	Rho GTPase activating protein 9	BB327418	10 D3	1436097_x_at	13.300	4.200	205.700	210.500	216.200
Usp18	ubiquitin specific peptidase 18	NM_011909	6 F	1418191_at	7.400	7.100	335.800	21.800	289.300
St8sia4	ST8 alpha-N-acetyl-neuraminide alpha-2,8-	NM_009183	1 D	1419186_a_at	30.200	14.300	612.300	907.200	1413.400
Lgals9	lectin, galactose binding, soluble 9	NM_010708	11 B5	1421217_a_at	69.300	61.200	2617.300	426.900	484.700
Mpo	myeloperoxidase	NM_010824	11 C	1415960_at	154.800	114.400	4844.898	624.900	16.300
Trim30	tripartite motif protein 30	BM240719	7 E3	1417961_a_at	18.800	5.400	228.300	766.800	924.200
		AF220015	7 E3	1451860_a_at	27.900	17.800	294.800	188.000	373.600
Adcy7	adenylate cyclase 7	BB746807		1456307_s_at	32.700	33.000	1293.800	1817.500	1072.300
		NM_007406	8 C3	1450065_at	16.900	10.700	417.700	261.000	217.500
Ifih1	interferon induced with helicase C domain 1	AY075132	2 C3	1426276_at	2.600	10.400	397.600	765.200	999.200

Ptgs1	prostaglandin-endoperoxide synthase 1	BB520073	2 B	1423414_at	20.400	5.900	224.700	256.300	326.600
		AA833146	2 B	1436448_a_at	13.000	14.300	206.900	351.300	365.500
Ifi47	interferon gamma inducible protein 47	NM_008330	11 B1.2	1417292_at	11.700	5.600	207.300	833.200	2423.100
Slamf1	signaling lymphocytic activation molecule family	BB132695	1 H3	1425570_at	10.200	14.300	522.700	223.200	143.400
Pla2g4a	phospholipase A2, group IVA (cytosolic, calcium-dependent)	NM_008869	1 G1	1448558_a_at	47.200	22.800	784.300	470.100	1298.600
Gvin1	GTPase, very large interferon inducible 1	BM243571	7 E3	1429184_at	12.500	19.200	660.000	2112.800	2077.300
Btk	Bruton agammaglobulinemia tyrosine kinase	NM_013482	X E3	1422755_at	1.200	15.000	513.200	222.500	350.900
Dock2	dedicator of cyto-kinesis 2	NM_033374	11 A5	1422808_s_at	12.400	10.300	331.700	1408.500	819.000
Ugt1a2	UDP glucuronosyltransferase 1 family, polypeptide A2	D87867	1 D ; 1 D	1426260_a_at	15.600	12.100	387.800	250.500	248.300
Lst1	leukocyte specific transcript 1	U72644	17 B1	1425548_a_at	9.000	18.100	578.800	998.700	534.700
Gbp6	guanylate binding protein 6	BM241271	3 H1	1434380_at	33.500	17.900	565.500	918.900	932.600
Dock10	dedicator of cytokinesis 10	BF715043	1 C5	1435343_at	9.400	9.000	278.000	521.400	427.900
6330500D04Rik	RIKEN cDNA 6330500D04 gene	BM242294	13 A3.2	1460555_at	11.500	9.600	280.600	980.700	470.700
Coro2a	coronin, actin binding protein 2A	AI316747	4 B1	1452367_at	12.900	7.400	213.900	220.900	232.000
Ankrd44	ankyrin repeat domain 44	AV256780	1 C1.2	1434856_at	23.500	9.000	246.000	208.700	185.100
AI586015	expressed sequence AI586015	NM_019992	5 E1	1421098_at	6.200	8.600	228.200	183.600	219.400
Parp9	poly (ADP-ribose) polymerase family, member 9	NM_030253	16 B3	1416897_at	20.500	10.800	272.200	441.900	832.800
Rgs2	regulator of G-protein signaling 2	AF215668	1 F	1419247_at	27.500	22.600	568.800	2383.900	23.500
			1 F	1419248_at	51.900	45.300	875.500	2819.800	105.300
	regulator of G-protein signaling 2	BB034265	1 F	1447830_s_at	24.100	16.200	286.300	1291.800	53.700
Bcl11a	B-cell CLL	NM_016707	11 A3.2	1419406_a_at	14.500	17.100	428.000	1133.900	555.600
H2-D1	histocompatibility 2, D region locus 1	L36068	17 B1	1451784_x_at	69.800	28.100	702.300	4307.000	3522.000
Centb1	centaurin, beta 1	BB115902	11 B3	1434873_a_at	30.700	30.500	731.400	1667.000	691.600
		BB757196	11 B3	1437117_at	34.300	30.700	321.800	497.800	382.600
Lcp1	lymphocyte cytosolic protein 1	NM_008879	14 D3	1448160_at	57.900	37.300	869.700	276.500	717.600
			14 D3	1415983_at	68.400	65.100	1272.000	2087.100	2622.100
Lyzs	lysozyme	AW208566	10 D2	1423547_at	27.100	18.600	419.700	942.200	233.700
Ly6e	lymphocyte antigen 6 complex, locus E	BM245572	15 D3	1453304_s_at	98.000	63.600	1408.400	2529.600	3664.900
Cdk6	cyclin-dependent kinase 6	BM238926	5 A2-A3	1455287_at	76.200	56.700	1227.900	703.700	919.900
			5 A2-A3	1435338_at	72.800	49.400	1046.300	639.300	767.600
H2-L	histocompatibility 2, D region	M69068	17 B1	1451931_x_at	56.500	26.900	564.600	4539.600	3575.800
H2-D1	histocompatibility 2, D region locus 1	M86502	17 B1	1425545_x_at	91.800	34.700	724.800	4509.600	3430.900
Gimap6	GTPase, IMAP family member 6	BB667753	6 B2.3	1427891_at	29.400	14.400	292.300	747.700	416.900
Ncf2	neutrophil cytosolic factor 2	NM_010877	1 G3	1448561_at	19.900	16.800	337.400	617.600	242.000
Clu	clusterin	AV152288	14 D1	1437689_x_at	141.200	57.800	1116.400	466.200	375.600
		BB433678	14 D1	1454849_x_at	145.700	90.000	1191.500	508.000	437.000
		AV075715	14 D1	1437458_x_at	110.000	79.500	974.100	496.200	433.000
Lrmp	lymphoid-restricted membrane protein	NM_008511	6 G3	1448409_at	22.600	15.900	296.100	300.700	200.200
Il2rg	interleukin 2 receptor, gamma chain	L20048	X C3	1416295_a_at	61.400	52.300	972.000	1599.100	2037.000
			X C3	1416296_at	48.500	39.000	643.400	796.500	1577.300
Mbnl3	muscleblind-like 3 (Drosophila)	AV306759	X A4	1434678_at	31.600	12.200	226.500	527.300	939.800
EG622147	predicted gene, EG622147	BB794742		1455892_x_at	11.800	16.900	302.800	526.200	1227.400
Cd1d1	CD1d1 antigen	NM_007639	3 F1	1449130_at	23.700	13.000	229.900	652.700	132.800
Cmtm7	CKLF-like MARVEL transmembrane domain	NM_133978	9 F3	1460253_at	80.400	80.800	1394.700	613.000	619.300
Myo1g	myosin IG	BB235320		1427892_at	15.600	14.700	250.200	364.500	359.800
Cugbp2	CUG triplet repeat, RNA binding protein 2	BB644164	2 A2-A3	1423895_a_at	38.700	25.400	415.700	1889.800	1553.400
			2 A2-A3	1451154_a_at	103.300	71.900	1046.000	1183.900	924.200
		BB667096	2 A2-A3	1450069_a_at	81.900	44.000	562.300	615.800	560.300
Akr1c12	aldo-keto reductase family 1, member C12	AF177041	13 A1	1450455_s_at	23.500	21.600	350.300	316.700	973.300
Akr1c13	aldo-keto reductase family 1, member C13	NM_013778	13 A1	1418672_at	28.200	14.300	228.400	557.500	1277.700

Fxyd5	FXYD domain-containing ion transport regulator 5	NM_008761	7 A3	1418296_at	24.400	27.000	416.400	2158.300	1669.100
Spn	sialophorin	BB160586	7 F3-F4	1439034_at	9.700	14.200	216.900	1568.200	1243.900
Rab38	Rab38, member of RAS oncogene family	NM_028238	7 D3	1417700_at	88.700	69.800	1057.400	1549.000	1688.300
		AV364767	7 D3	1439628_x_at	71.500	72.400	899.500	2652.400	2559.400
Emilin2	elastin microfibril interfacer 2	BB811788	17 E1.3	1435264_at	54.500	54.700	826.700	36.500	307.800
Mpl	myeloproliferative leukemia virus oncogene	NM_010823	4 D	1421461_at	31.700	23.800	357.300	1965.300	883.600
Rgs1	regulator of G-protein signaling 1	NM_015811	1 F	1417601_at	16.000	17.600	246.200	2592.200	147.500
Pycard	PYD and CARD domain containing	BG084230	7 F4	1417346_at	62.700	44.000	609.500	256.900	932.100
---	---	AW556821	X A3.3	1419033_at	82.600	57.100	776.700	1744.300	1538.800
Lrrc33	leucine rich repeat containing 33	BC027411	16 B2	1451174_at	63.100	58.600	794.000	471.700	996.400
D11Ertd759e	DNA segment, Chr 11, ERATO Doi 759, expressed	AW556558	11 E2	1455500_at	47.000	47.800	641.600	701.300	1209.300
1200013B08Rik	RIKEN cDNA 1200013B08 gene	AK004734	X A4	1427007_at	43.200	43.000	544.300	955.700	621.700
Plek	pleckstrin	AF181829	11 A2	1448748_at	184.800	119.500	1505.400	609.000	168.600
Dtx3l	deltex 3-like (Drosophila)	AV327407	16 B3	1435208_at	33.100	22.900	282.300	444.200	513.600
Ptger3	prostaglandin E receptor 3 (subtype EP3)	NM_011196	3 H4	1450344_a_at	35.900	29.400	354.600	571.600	666.400
Klk8	kallikrein related-peptidase 8	NM_008940	7 B3	1419722_at	33.100	24.500	292.900	322.300	86.300
Nckap1l	NCK associated protein 1 like	BM238906	15 F3	1428786_at	12.000	22.700	268.500	753.300	512.900
Fos	FBJ osteosarcoma oncogene	AV026617	12 D2	1423100_at	341.400	130.300	1480.500	6066.600	14.100
AW491445	expressed sequence AW491445	AV300228	19 A	1426883_at	25.800	19.100	215.200	625.300	389.300
Casp8	caspase 8	BC006737	1 B	1424552_at	61.000	40.900	455.900	450.400	827.700
Pik3cg	phosphoinositide-3-kinase, catalytic, gamma polypeptide	BB205102	12 B	1422707_at	17.100	25.800	278.400	234.000	350.500
Ifitm1	interferon induced transmembrane protein 1	BC027285	7 F5	1424254_at	308.100	235.700	2539.301	5467.600	4116.800
Vamp5	vesicle-associated membrane protein 5	AK009266	6 C1	1430522_a_at	22.900	23.200	249.900	653.700	1413.300
Stat1	signal transducer and activator of transcription 1	AW214029	1 C1.1	1450033_a_at	51.500	27.800	294.000	242.300	747.100
			1 C1.1	1450034_at	53.800	39.100	389.700	440.600	900.200
Samsn1	SAM domain, SH3 domain and nuclear localization signals, 1	NM_023380	16 C3.1	1421457_a_at	112.100	82.800	860.100	373.500	478.400
Mylk	myosin, light polypeptide kinase	BF451748	16 B3	1425506_at	41.500	24.500	242.900	398.700	243.200
Irgm	immunity-related GTPase family, M	NM_008326	11 B1.2	1418825_at	22.800	28.500	282.400	241.400	606.900
Birc3	baculoviral IAP repeat-containing 3	NM_007464	9 A2	1421392_a_at	64.300	29.200	287.600	864.000	189.800
Nr4a1	nuclear receptor subfamily 4, group A, member 1	NM_010444	15 F	1416505_at	84.100	65.300	632.300	5624.100	35.600
Csf2rb2	colony stimulating factor 2 receptor, beta 2, low-affinity	NM_007781	15 E1	1449360_at	70.300	74.900	713.000	110.900	375.400
Lgals1	lectin, galactose binding, soluble 1	NM_008495	15 E	1419573_a_at	873.400	425.800	4024.000	203.800	471.500
		AI642438	15 E	1455439_a_at	1521.600	731.400	6338.198	414.100	806.800
Cyfip2	cytoplasmic FMR1 interacting protein 2	AK005148		1428347_at	27.700	23.700	219.900	648.300	299.000
Icam1	intercellular adhesion molecule	BC008626	9 A3	1424067_at	56.400	25.200	231.400	1474.400	163.800
AY078069	cDNA sequence AY078069	AW495632	7 A3	1436074_at	29.300	23.700	214.500	1186.200	138.000
Foxp1	forkhead box P1	BM220880	6 E1	1435222_at	67.200	52.300	472.400	689.300	185.800
		BG962849	6 E1	1421140_a_at	48.600	36.600	251.400	347.400	85.700
Elf1	E74-like factor 1	NM_007920	14 D3	1417540_at	98.200	78.900	698.300	616.800	321.300
Laptm5	lysosomal-associated protein transmembrane 5	BB218107	4 D2.3	1436905_x_at	238.800	190.700	1666.900	4201.000	2986.000
		U29539	4 D2.3	1426025_s_at	70.100	91.300	564.600	1488.700	567.800
Chst11	carbohydrate sulfotransferase 11	AK003880	10 C1	1428902_at	36.500	46.200	403.100	314.800	71.200
Rbp1	retinol binding protein 1, cellular	NM_011254	9 E3.3	1448754_at	66.900	30.300	250.700	542.200	171.200
Csf2rb1	colony stimulating factor 2 receptor, beta 1, low-affinity	BB769628		1455660_at	355.600	285.000	2343.300	86.000	492.900
Muc13	mucin 13, epithelial transmembrane	NM_010739	16 B3	1419387_s_at	86.400	57.000	464.400	1068.800	1759.000
Ptpn18	protein tyrosine phosphatase, non-receptor type 18	NM_011206	1 B	1419125_at	92.600	71.400	580.400	849.700	495.300
Nfkbia	nuclear factor of kappa light chain gene enhancer in B-cells	BB096843	12 C1-C3	1438157_s_at	508.000	203.400	1623.800	5997.100	1381.200
		NM_010907	12 C1-C3	1448306_at	188.900	85.200	451.200	2915.800	245.400
4632417K18Rik	RIKEN cDNA 4632417K18 gene	NM_026640	19 B	1422628_at	110.600	101.100	801.700	208.400	1302.000
Nfkbiz	nuclear factor of kappa light polypeptide gene enhancer in	AB026551	16 C1.2-C1.3	1417483_at	98.900	43.700	343.500	3405.100	114.300

Sec11c	SEC11 homolog C (S. cerevisiae)	AK007641	18 E1	1460698_a_at	100.500	111.200	820.500	658.200	755.500
D330050I23Rik	RIKEN cDNA D330050I23 gene	BE303700	15 D1	1434301_at	34.100	34.600	255.200	70.800	241.300
LOC677168	hypothetical protein LOC677168	AK019325	17 B3	1431591_s_at	32.400	27.700	202.700	78.200	265.000
Plscr1	phospholipid scramblase 1	BF319989	9 E3.3 ; 1 C5	1429527_a_at	111.800	78.400	571.200	249.300	868.000
Tapbp	TAP binding protein	AF043943	17 B1	1421812_at	91.600	79.700	579.600	1393.700	2514.700
Emb	embigin	BG064842	13 D2.3	1415857_at	662.400	309.500	2231.600	312.700	550.300
		BG064842	13 D2.3	1415856_at	265.400	146.600	800.400	379.400	562.000
Plscr1	phospholipid scramblase 1	BF319989	9 E3.3	1453181_x_at	119.800	105.900	762.400	357.700	1462.800
Lgals3bp	lectin, galactoside-binding, soluble, 3 binding protein	NM_011150	11 E	1448380_at	51.700	57.600	408.000	658.300	1253.500
Arhgdib	Rho, GDP dissociation inhibitor (GDI) beta	AK002516	6 G1	1426454_at	535.300	432.700	3019.300	1714.300	1217.000
Socs3	suppressor of cytokine signaling 3	BB241535	11 E2	1455899_x_at	56.800	35.400	244.300	1882.600	66.900
Nfkbia	nuclear factor of kappa light chain gene enhancer in B-cells	AI462015		1449731_s_at	141.100	72.000	495.200	2576.300	431.700
Stard10	START domain containing 10	NM_019990	7 E1	1448956_at	56.900	55.400	380.300	540.700	714.000
Ripk3	receptor-interacting serine-threonine kinase 3	NM_019955	14 C1	1448449_at	25.400	29.800	201.200	673.500	1065.300
Tspo	translocator protein	AV101079	15 E2	1438948_x_at	73.000	51.100	339.300	599.800	866.600
H2-T23	histocompatibility 2, T region locus 23	NM_010398	17 B1	1449556_at	33.600	46.000	295.200	3047.500	2498.300
Mgst1	microsomal glutathione S-transferase 1	BI150149	6 G1	1415897_a_at	109.600	77.400	495.600	349.200	92.100
Brd4	bromodomain containing 4	BC008532	8 B3.3	1424921_at	43.100	44.100	280.600	126.300	873.000
Tspo	translocator protein	BC002055	15 E2	1416695_at	109.600	84.000	531.500	340.100	977.600
Basp1	brain abundant, membrane attached signal protein 1	AK011545	15 B1	1428572_at	309.800	111.700	704.600	777.600	1645.400
Cd34	CD34 antigen	NM_133654	1 H6	1416072_at	216.100	146.800	925.100	529.200	678.000
3110001A13Rik	RIKEN cDNA 3110001A13 gene	BC021353	2 A1	1416892_s_at	226.700	123.700	776.000	5144.000	2071.300
			2 A1	1416893_at	112.500	67.500	354.500	532.600	54.100
Ifi35	interferon-induced protein 35	AW986054	11 D	1459151_x_at	46.800	35.100	217.200	252.400	711.300
			11 D	1445897_s_at	197.300	170.400	933.300	1316.200	2230.900
Itga6	integrin alpha 6	BM935811	2 C2	1422444_at	89.400	51.200	316.200	241.600	219.200
Isgf3g	interferon dependent positive acting transcription factor 3	NM_008394	14 C2	1421322_a_at	50.300	40.100	244.100	192.400	237.600
Man2a2	mannosidase 2, alpha 2	BB794673	7 D2	1435203_at	66.900	61.000	371.100	933.500	770.900
Nt5c	5',3'-nucleotidase, cytosolic	NM_015807	11 E	1417252_at	103.600	62.600	371.700	177.600	548.800
Myd88	myeloid differentiation primary response gene 88	BC005591	9 F3	1419272_at	43.800	42.700	253.100	153.400	367.800
Elmo1	engulfment and cell motility 1, ced-12 homolog (C. elegans)	BC024727	13 A3.1	1424523_at	40.900	41.500	245.900	295.200	367.900
Tgfbr2	transforming growth factor, beta receptor II	BG793483	9 F3	1426397_at	177.100	114.300	675.600	647.500	466.900
Igtp	interferon gamma induced GTPase	NM_018738	11 B1.3	1417141_at	23.100	42.100	248.300	688.600	3013.700
Rcsd1	RCSD domain containing 1	BC025872	1 H2.3	1424194_at	39.800	35.100	205.600	618.700	339.100
Cyba	cytochrome b-245, alpha polypeptide	AK018713		1454268_a_at	183.100	129.300	755.100	706.500	895.000
Nr3c1	nuclear receptor subfamily 3, group C, member 1	NM_008173	18 B3	1460303_at	37.600	35.800	206.700	369.800	232.700
5033414D02Rik	RIKEN cDNA 5033414D02 gene	BC024953	19 C1	1460361_at	169.500	139.600	783.500	532.500	353.500
Acsl5	acyl-CoA synthetase long-chain family member 5	AK006541	19 D2	1428082_at	166.100	194.200	1088.200	1296.600	1556.600
Tspo	translocator protein	BB132602	15 E2	1456251_x_at	44.400	49.400	275.200	319.300	623.100
---	Transcribed locus	BG143461		1436570_at	58.100	49.100	273.000	473.400	410.500
Osbpl8	oxysterol binding protein-like 8	BG969333	10 D1	1437069_at	126.800	80.500	442.200	225.100	176.800
Tes	testis derived transcript	BC010465	6 A2	1460378_a_at	107.100	57.200	312.500	374.000	604.200
Ctsc	cathepsin C	NM_009982	7 D3-E1.1	1416382_at	217.800	193.000	1043.400	899.800	1579.700
Gsn	gelsolin	AV224521	2 B	1456312_x_at	202.400	110.000	590.900	128.300	320.000
Fut8	fucosyltransferase 8	NM_016893	12 C3	1460319_at	453.900	266.900	1432.100	821.200	1852.700
B3gnt2	UDP-GlcNAc:betaGal beta-1,3-N-	AV306734	11 A3.2	1450026_a_at	125.200	120.000	640.500	402.600	255.200
Traf3	Tnf receptor-associated factor 3	U21050	12 F	1418587_at	70.800	45.300	241.100	345.500	395.100
Itga4	integrin alpha 4	BB205589	2 C3	1436037_at	80.000	98.800	525.600	431.200	245.100
Nfkbia	nuclear factor of kappa light chain gene enhancer in B-cells	AI462015		1420088_at	1100.500	521.400	2713.800	6326.200	2784.800
Lyn	Yamaguchi sarcoma viral (v-yes-1) oncogene homolog	M57697	4 A1	1451318_a_at	136.600	89.700	459.700	495.300	309.700

Gusb	glucuronidase, beta	NM_010368	5 G1.3	1448124_at	138.500	175.200	892.300	272.600	1145.100
B2m	beta-2 microglobulin	BF715219	2 F1-F3	1449289_a_at	610.500	571.100	2860.500	5307.900	4332.300

After ESCs differentiated into EB6 cells without HOXB4 expression, c-Kit+CD41+ cells were isolated (EB6). These cells were co-cultured with OP9 cells for 4 days, with or without HOXB4 expression, followed by recovery of c-Kit+CD41+ cells from the co-cultures (HOXB4-off and HOXB4-on). CD34-KSL cells were isolated from adult bone marrow cells. Since most freshly isolated CD34-KSL cells are in hibernation (hibernating HSCs), to obtain cycling HSCs, CD34-KSL cells were cultivated in the presence of stem cell factor and TPO for 24 hours (cycling HSCs). RNAs extracted from these samples were used for microarray analysis. To increase the reliability of data, probes with gene chip scores > 200 in HOXB4-off or HOXB4-on data were used for comparison. The HOXB4-on : HOXB4-off ratio of >5.0 was used for selection of up-regulated genes. To see if up-regulated genes include genes expressed in adult HSCs, probes with gene chip scores >200 were extracted from data for hibernating and cycling HSCs and used for comparison.
