# Supplementary material for: Stepwise Development of Hematopoietic Stem Cells from Embryonic Stem Cells
Source: PLoS One. 2009 Mar 16;4(3):e4820. doi: 10.1371/journal.pone.0004820 (PMC2653650; doi:10.1371/journal.pone.0004820)
Supplement: Table S4 — (0.06 MB PDF) [file pone.0004820.s004.pdf]

Table S4 Adult HSC-unrelated genes down-regulated by HOXB4

Gene	Gene Title	Genbank	Map	MOE430v2	EB6	HOXB4-off	HOXB4-on	Hibernating HSC	Cycling HSCs
Slc32a1	solute carrier family 32 (GABA vesicular transporter), member 1	NM_009508	chr2 H1-3	1422756_at	214.200	260.600	1.200	1.100	1.000
				1419592_at	178.500	210.200	1.200	3.300	3.800
Hba-x	hemoglobin X, alpha-like embryonic chain in Hba complex	M26898	chr11 A4|11 16.0 cM	1448716_at	6059.601	6028.199	41.100	3.500	0.800
Lin28	lin-28 homolog (C. elegans)	BB706377	chr4 D3	1437752_at	363.800	357.600	5.400	0.400	3.100
Alas2	aminolevulinic acid synthase 2, erythroid	M63244	chrX F3|X 63.0 cM	1451675_a_at	451.500	537.200	10.600	0.500	0.400
		NM_008007	chr7 F5|7 72.4 cM	1422923_at	147.800	246.800	1.200	4.200	8.700
		AV302620	---	1441350_at	265.400	503.500	6.700	7.000	1.400
Fgf3	fibroblast growth factor 3	BB391920	chr7 F5|7 72.4 cM	1441914_x_at	139.500	201.600	4.100	1.900	2.300
Trim10	tripartite motif protein 10	NM_011280	chr17 B1	1419311_at	243.600	303.700	9.800	7.900	2.900
Hand2	---	BB029192	---	1436041_at	337.000	315.000	12.000	3.900	0.200
		BB036951	chr13 C3	1438020_at	357.200	279.900	9.400	0.700	0.500
Hapln1	hyaluronan and proteoglycan link protein 1	AF098460	chr13 C3	1426294_at	246.200	211.500	9.300	0.200	2.400
Epb4.2	erythrocyte protein band 4.2	U03487	chr2 E5|2 67.5 cM	1417337_at	137.800	224.500	9.900	14.000	2.400
D130058I21Rik	RIKEN cDNA D130058I21 gene	BB458758	chr11 B4	1455794_at	478.400	530.600	24.500	13.800	5.800
		AV147727	chr7 E3|7 49.96 cM	1437990_x_at	11821.904	10749.297	308.000	0.500	0.400
		AV311770	chr7 E3|7 49.96 cM	1437810_a_at	10115.196	9833.396	302.300	7.700	12.900
Hbb-bh1	hemoglobin Z, beta-like embryonic chain	NM_008219	chr7 E3|7 49.96 cM	1450736_a_at	9369.304	8375.498	411.000	1.100	0.400
		NM_008221	chr7 E3|7 49.95 cM	1450621_a_at	1637.000	1144.200	35.800	0.400	0.800
		AV148191	chr7 E3|7 49.95 cM	1436823_x_at	4416.398	3291.301	162.400	3.100	49.200
Hbb-y	hemoglobin Y, beta-like embryonic chain	AV156860	chr7 E3|7 49.95 cM	1436717_x_at	4365.399	3295.899	163.200	3.200	46.800
Rragd	Ras-related GTP binding D	BF462770	chr4 A5|4 11.4 cM	1434909_at	339.700	302.300	15.500	18.000	3.000
Slc30a10	solute carrier family 30, member 10	BB540543	chr1 H5	1439934_at	215.000	246.200	13.300	8.000	3.000
Slc39a8	solute carrier family 39 (metal ion transporter), member 8	NM_026228	chr3 G3|3 66.0 cM	1448482_at	650.100	638.800	35.200	3.900	6.400
		M26385	chr8 C2|8 36.0 cM	1425643_at	602.400	840.900	42.200	1.200	1.000
Gypa	glycophorin A	NM_010369	chr8 C2|8 36.0 cM	1423016_a_at	703.200	971.800	55.500	0.200	0.500
Reln	reelin	NM_011261	chr5 A3-B1|5 8.0 cM	1449465_at	1978.700	2389.501	141.700	7.600	4.700
Unc5c	unc-5 homolog C (C. elegans)	NM_009472	chr3 H1|3 68.5 cM	1449522_at	280.900	301.800	18.200	17.500	11.300
Capn6	calpain 6	AI747133	chrX F2	1450429_at	335.300	206.800	13.300	1.100	3.700
Rspo3	R-spondin 3 homolog (Xenopus laevis)	BG072958	chr10 A4	1455607_at	376.000	477.200	34.000	3.200	4.000
Sox11	SRY-box containing gene 11	BG072739	chr12 A3	1436790_a_at	237.600	215.700	16.100	0.200	7.500
Dp1l1	receptor accessory protein 6	AK002562	chr10 C1	1430128_a_at	264.500	325.000	25.700	22.900	32.000
Mdk	midkine	M34328	chr2 E1|2 53.0 cM	1416006_at	3244.600	3309.301	265.000	46.300	13.800
Tspan12	tetraspanin 12	BB072896	chr6 A3.1	1454604_s_at	188.500	321.900	26.800	6.400	1.200
Igfbp5	insulin-like growth factor binding protein 5	BF225802	chr1 C3|1 36.1 cM	1452114_s_at	413.800	334.000	27.900	0.900	0.500
Nasp	nuclear autoantigenic sperm protein (histone-binding)	BB493242	chr4 56.5 cM	1416043_at	409.500	444.800	37.900	1.000	2.400
Fn1	fibronectin 1	BM234360	chr1 C1-C5|1 36.1 cM	1437218_at	339.000	363.400	34.600	1.400	6.100
		BE283964	chr3 H3	1429298_at	628.400	799.200	25.000	3.400	8.300
		BB770857	chr3 H3	1455400_at	180.700	241.600	9.000	1.000	6.300
Ddah1	dimethylarginine dimethylaminohydrolase 1	AW556888	chr3 H3	1454995_at	282.200	296.600	32.200	15.500	14.800
		NM_008218	chr11 A4|11 16.0 cM	1417714_x_at	776.600	841.900	43.600	0.500	0.500
		AK011116	chr11 A4|11 16.0 cM	1452757_s_at	7352.003	6973.301	763.300	10.000	9.800
Hba-a1	hemoglobin alpha, adult chain 1	AK011116	chr11 A4|11 16.0 cM	1428361_x_at	7497.502	7305.399	832.900	12.500	1.700
Sall4	sal-like 4 (Drosophila)	BG064756	chr2 H3|2 99.0 cM	1424152_at	363.800	299.500	37.000	7.300	0.500
Enpp1	ectonucleotide pyrophosphatase/phosphodiester	AF339910	chr10 A4|10 19.0 cM	1419276_at	268.000	303.400	38.100	23.300	12.700
B230112C05Rik	RIKEN cDNA B230112C05 gene	BB476773	chr13 D1	1435783_at	211.900	250.800	31.500	12.400	6.500
		NM_009221	chr6 B3|6 29.0 cM	1418493_a_at	282.700	403.700	44.700	12.000	9.300
Snca	synuclein, alpha	AI324124	chr6 B3|6 29.0 cM	1436853_a_at	674.200	894.800	127.800	15.000	17.400
Agtrl1	angiotensin receptor-like 1	BB483357	chr2 E1	1438651_a_at	1695.800	1213.100	175.400	2.500	2.200

Slc38a5	solute carrier family 38, member 5	BG066984	chrX A1.1	1454622_at	244.000	294.900	44.000	8.100	0.700
Eraf	erythroid associated factor	NM_133245	---	1449077_at	285.800	331.600	51.000	40.200	3.600
Podxl	podocalyxin-like	AF290209	chr6 A3.3|6 10.0 cM	1417396_at	256.700	233.900	36.000	1.800	12.400
Cth	cystathionase (cystathionine gamma-lyase)	BC019483	chr3 H4	1426243_at	252.700	390.200	62.300	11.800	13.600
2010011I20Rik	RIKEN cDNA 2010011I20 gene	AK008190	chr2 H3	1424695_at	196.800	245.200	39.300	11.400	26.700
Igsf4a	immunoglobulin superfamily, member 4A	NM_018770	chr9 B-C	1417378_at	189.800	209.800	36.600	32.600	16.200
		BB130087	chr9 D	1456329_at	532.500	550.100	86.100	4.400	0.800
A230098A12Rik	protogenin homolog (Gallus gallus)	BB295128	chr9 D	1438410_at	275.300	341.400	60.200	3.200	9.600
Gldc	glycine decarboxylase	NM_138595	chr19 C|19 25.0 cM	1416049_at	269.400	326.600	58.000	2.100	7.200
Abtb2	ankyrin repeat and BTB (POZ) domain containing 2	BB621938	chr2 E2	1433453_a_at	170.300	217.500	40.800	47.800	22.900

After ESCs differentiated into EB6 cells without HOXB4 expression, c-Kit+CD41+ cells were isolated (EB6). These cells were co-cultured with OP9 cells for 4 days, with or without HOXB4 expression, followed by recovery of c-Kit+CD41+ cells from the co-cultures (HOXB4-off and HOXB4-on). CD34-KSL cells were isolated from adult bone marrow cells. Since most freshly isolated CD34-KSL cells are in hibernation (hibernating HSCs), to obtain cycling HSCs, CD34-KSL cells were cultivated in the presence of stem cell factor and TPO for 24 hours (cycling HSCs). RNAs extracted from these samples were used for microarray analysis. To increase the reliability of data, probes with gene chip scores > 200 in HOXB4-off or HOXB4-on data were used for comparison. The HOXB4-on : HOXB4-off ratio of <0.2 was used for down-regulated genes. To see if down-regulated genes include genes not expressed in adult HSCs, probes with gene chip scores <50 were extracted from data for hibernating and cycling HSCs and used for comparison.
