# Supplementary material for: Stepwise Development of Hematopoietic Stem Cells from Embryonic Stem Cells
Source: PLoS One. 2009 Mar 16;4(3):e4820. doi: 10.1371/journal.pone.0004820 (PMC2653650; doi:10.1371/journal.pone.0004820)
Supplement: Table S5 — (0.03 MB PDF) [file pone.0004820.s005.pdf]

Table S5 PCR primers

Genes	Forward	Reverse
m*HoxB4*	5'-TGGATGCGCAAAGTTCACGTG-3'	5'-ATCTTGGTGTTGGGCAACTTG-3'
h*HOXB4*	5'-AGAACCCCCTGCACCCCA-3'	5'-AGAACCCCCTGCACCCCA-3'
Runx1	5'-TCCTATGACCAGTCCTACCAG-3'	5'-CCGTAGTATAGATGGTAGGAG-3'
Gata1	5'-AAAGATGGAATCCAGACGAGG-3'	5'-GTCAAGGCTATTCTGTGTACC-3'
Gata2	5'-AGTGCATGCAAGAGAAGTCAC-3'	5'-ATGGCAGTCACCATGCTGGAC-3'
Hbb-bh1	5'-AGTCCCCATGGAGTCAAAGA-3	5'-CTCAAGGAGACCTTTGCTCA-3'
Hbb-b1	5'-CTGACAGATGCTCTCTTGGG-3'	5'-CACAACCCCAGAAACAGACA-3'
Scl	5'-TGAAGATGGCACGGTCTTCTC-3'	5'-ATGGGAGAAAGGCAAGGCAG-3'
Brachyury	5'-TGCAGTCCATGATAACTGG-3'	5'-TACTGGCTGTCAGAAATGTC-3'
Flk1	5'-ATCTCCAGAACAGTAAGCGAAA-3'	5'-TCCCTGAGTCAGCGTGAA-3'
Gapdh	5'-CTTCACCACCATGGAGAAGGC-3'	5'-GGCATGGACTGTGGTCAT-3'

PCR primers used in this study are listed.
