# Supplementary figures and images for: Stepwise Development of Hematopoietic Stem Cells from Embryonic Stem Cells
Source: PLoS One. 2009 Mar 16;4(3):e4820. doi: 10.1371/journal.pone.0004820 (PMC2653650; doi:10.1371/journal.pone.0004820)

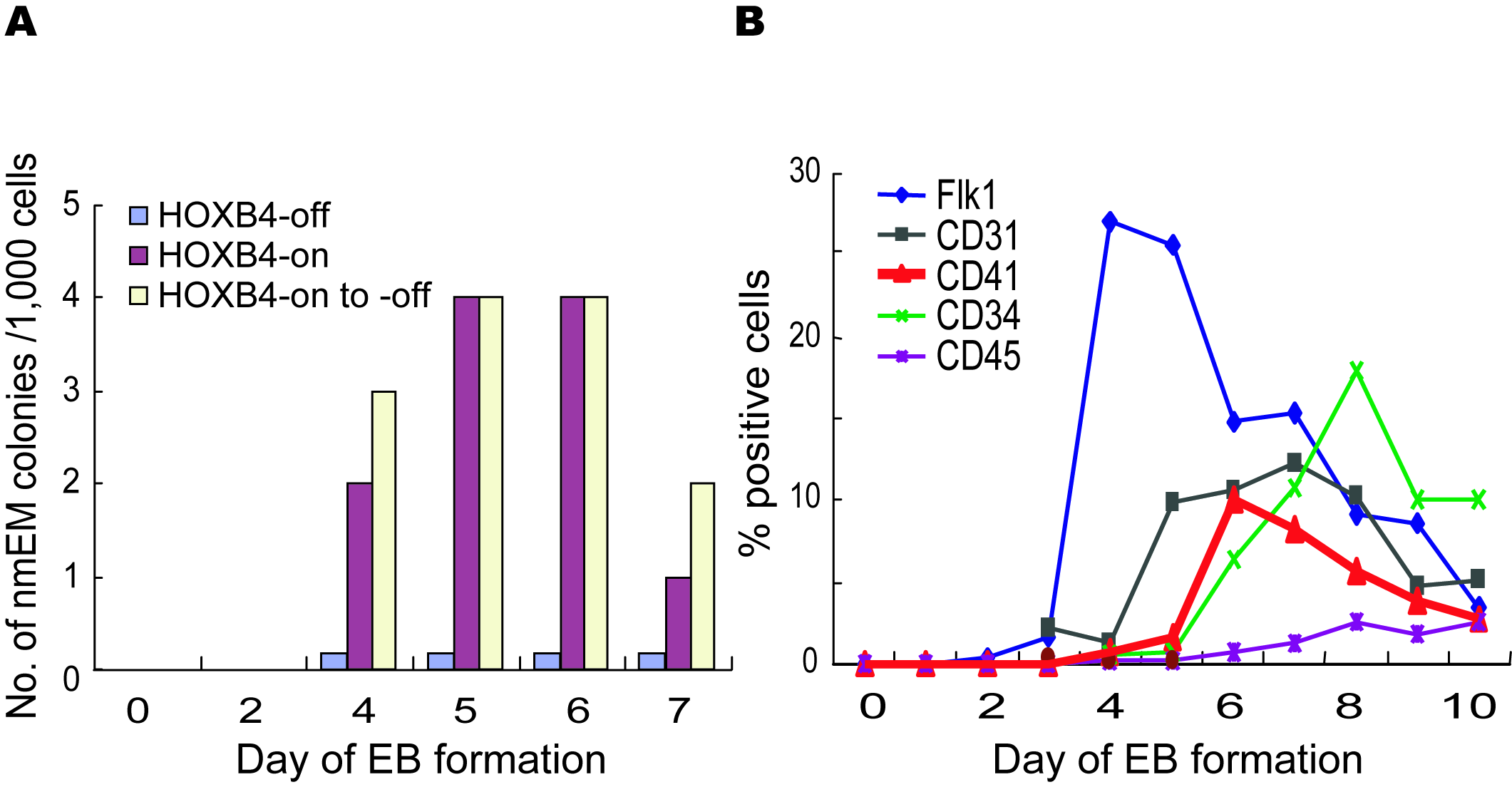

Supplement: Figure S1 — Effect of HOXB4 expression in generation of CFU-nmEM and surface marker expression. (A) Data show colony formation by 1,000 cells derived from EB. 1,000 cells were obtained from EBs on days 0, 2, 4, 5, 6, and 7 of culture and co-cultured with OP9 cells for 4 days. Cells collected from the co-cultures were plated in methylcellulose. HOXB4 expression was turned off in both OP9 co-cultures and methylcellulose cultures (HOXB4-off), turned on in both (HOXB4-on), or turned on only in OP9 co-cultures (HOXB4-on to -off). The number of colonies was counted and cells composing each colony were morphologically examined. The graphs show the numbers of neutrophil/macrophage/erythroblasts/megakaryocyte (nmEM) colonies extracted from various colonies formed. Continuous expression of HOXB4 in methylcellulose culture was not necessary for nmEM colony formation. The number of colonies is the mean from 2 independent experiments. (B) Cell surface markers were examined during EB formation without HOXB4 expression. Flk-1+ cells were first detected on day 3 of culture; the proportion of Flk-1+ cells markedly increased on the following day and decreased thereafter. CD31+ cells and CD41+ cells became detectable by day 5 and day 6, respectively, followed by the appearance of CD34+ cells. CD45+ cells became detectable after day 6, but remained low in number. c-Kit+ cells constituted about half of the cells throughout EB formation regardless of whether HOXB4 expression was induced (data not shown). (9.39 MB TIF) [file pone.0004820.s006.tif]

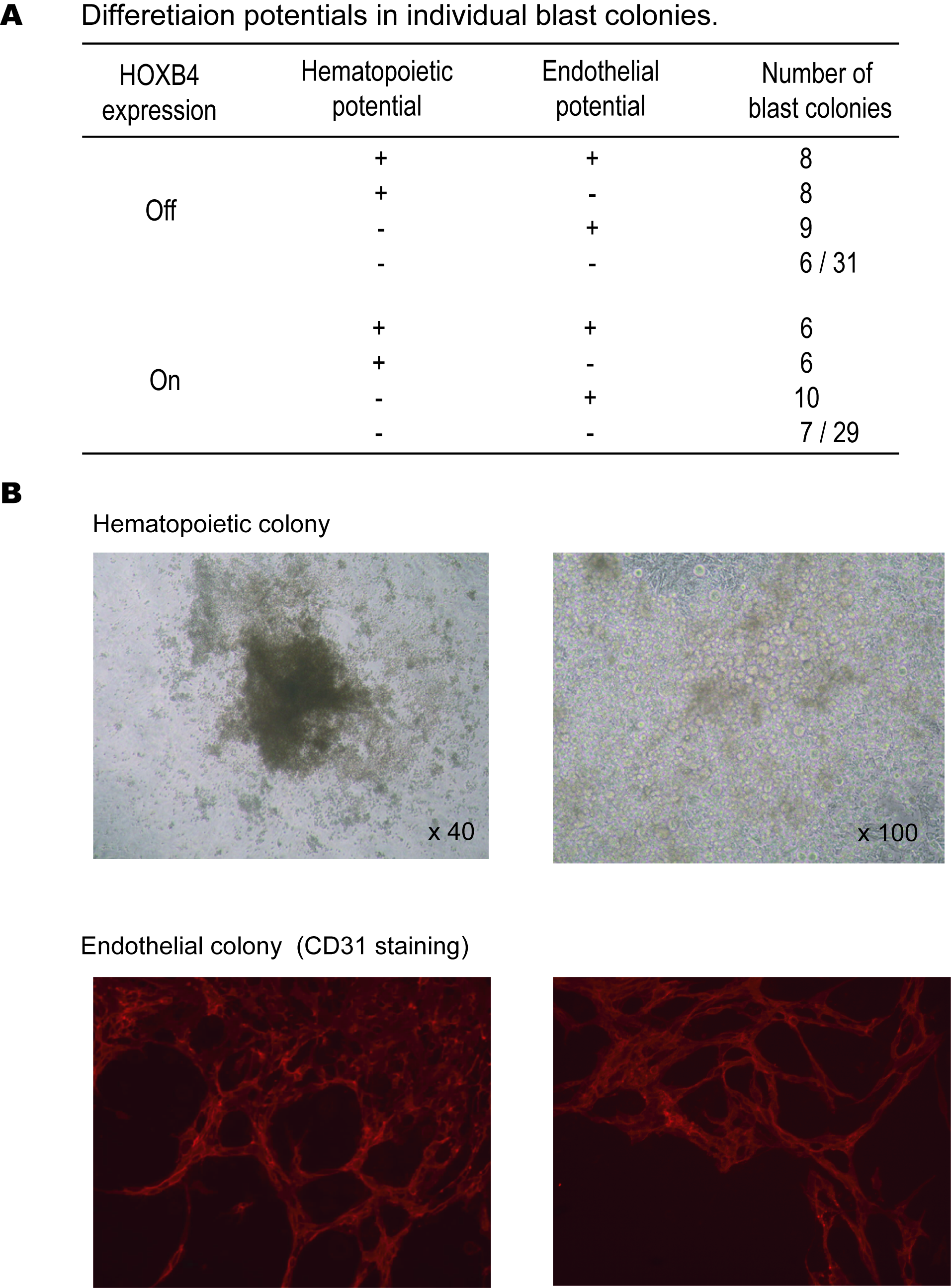

Supplement: Figure S2 — Differentiation potential of individual blast colonies. Both hematopoietic and endothelial potentials were examined for individual blast colonies. Blast colonies were formed by whole EB6 cells (Fig. 3A). Colonies were individually picked up from methylcellulose and co-cultured with OP9 cells in the presence of vascular endothelial growth factor, stem cell factor, interleukin-3, TPO, and erythropoietin for 7 days. (A) The summary of hematopoietic and endothelial potentials detected in individual blast colonies. (B) Representative photomicrographs show that a colony consisted of blood cells and CD31-positive vascular endothelial cells. (6.61 MB TIF) [file pone.0004820.s007.tif]

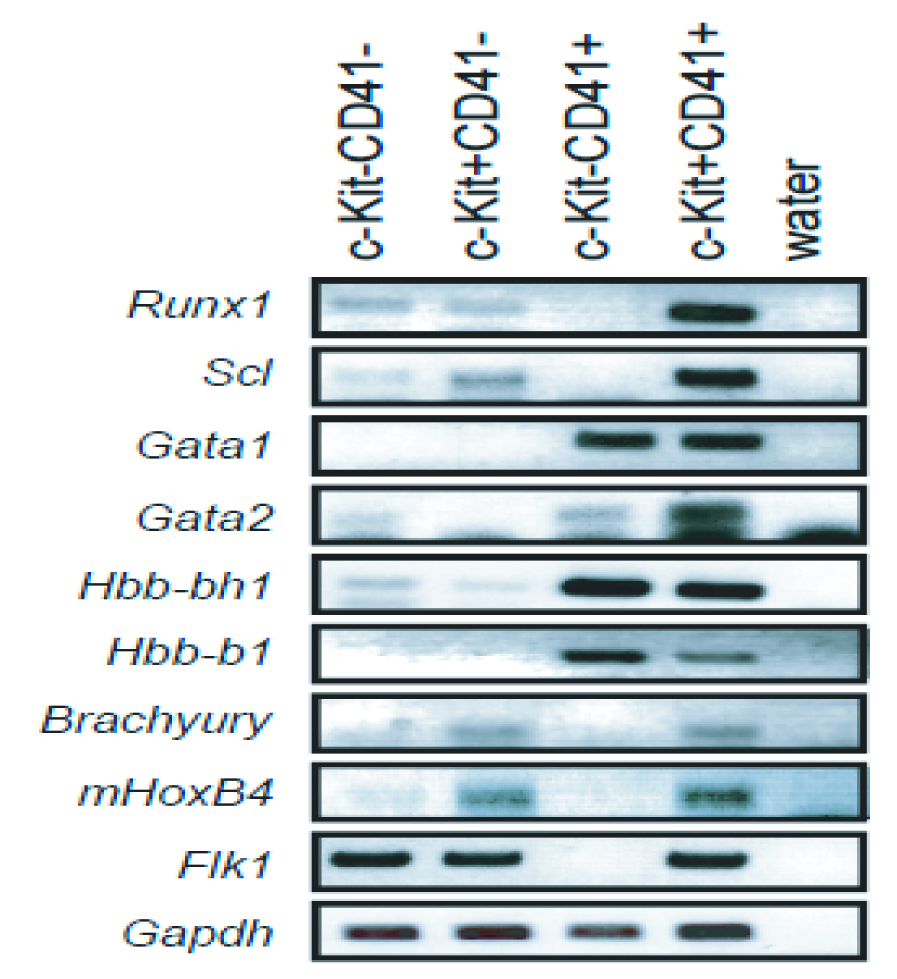

Supplement: Figure S3 — RT-PCR for 4 EB6 cell populations. PCR was performed on cDNAs prepared from fractionated EB6 cells. (3.55 MB TIF) [file pone.0004820.s008.tif]

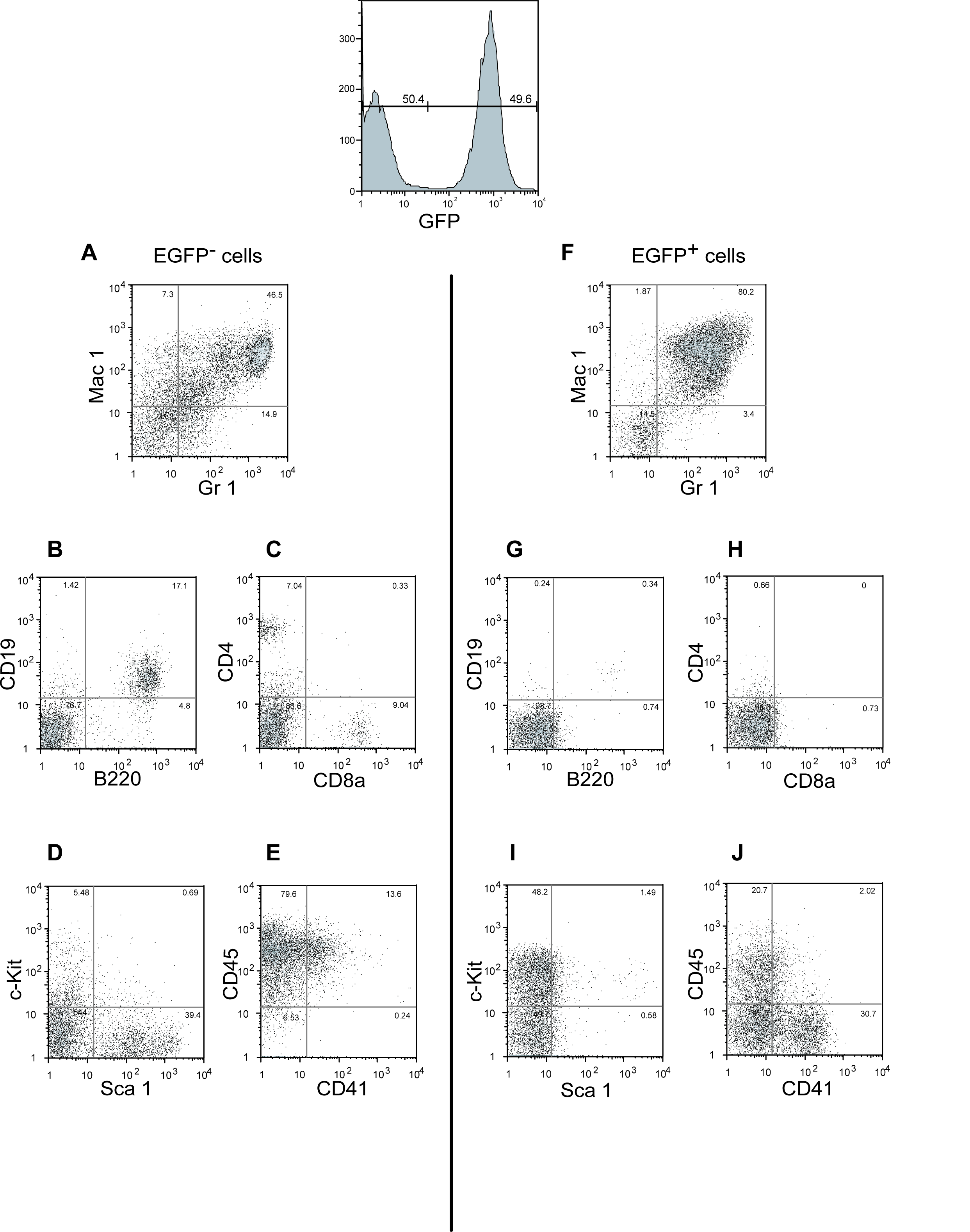

Supplement: Figure S4 — Analysis of bone marrow cells from recipient mice of HOXB4-expressing ES-derived cells. c-Kit+CD41+ EB6 cells were co-cultured with OP9 cells while HOXB4 was enforcedly expressed. After co-culture with OP9 cells, GFP+ cells and rescue cells were transplanted into lethally irradiated mice. 18 weeks after transplantation, bone marrow cells of the recipient mice were stained with antibodies and analyzed on a flow cytometer. (A–E) GFP− cells were derived from rescue cells and possibly from host cells. (F–J) GFP+ cells were derived from ESCs. GFP− cells and GFP+ cells are separately displayed for the expression of Gr-1 and Mac-1 (A, F), B220 and CD19 (B, G), CD4 and CD8 (C, H), Sca-1 and c-Kit (D, I), and CD41 and CD45 (E, J). (1.28 MB TIF) [file pone.0004820.s009.tif]

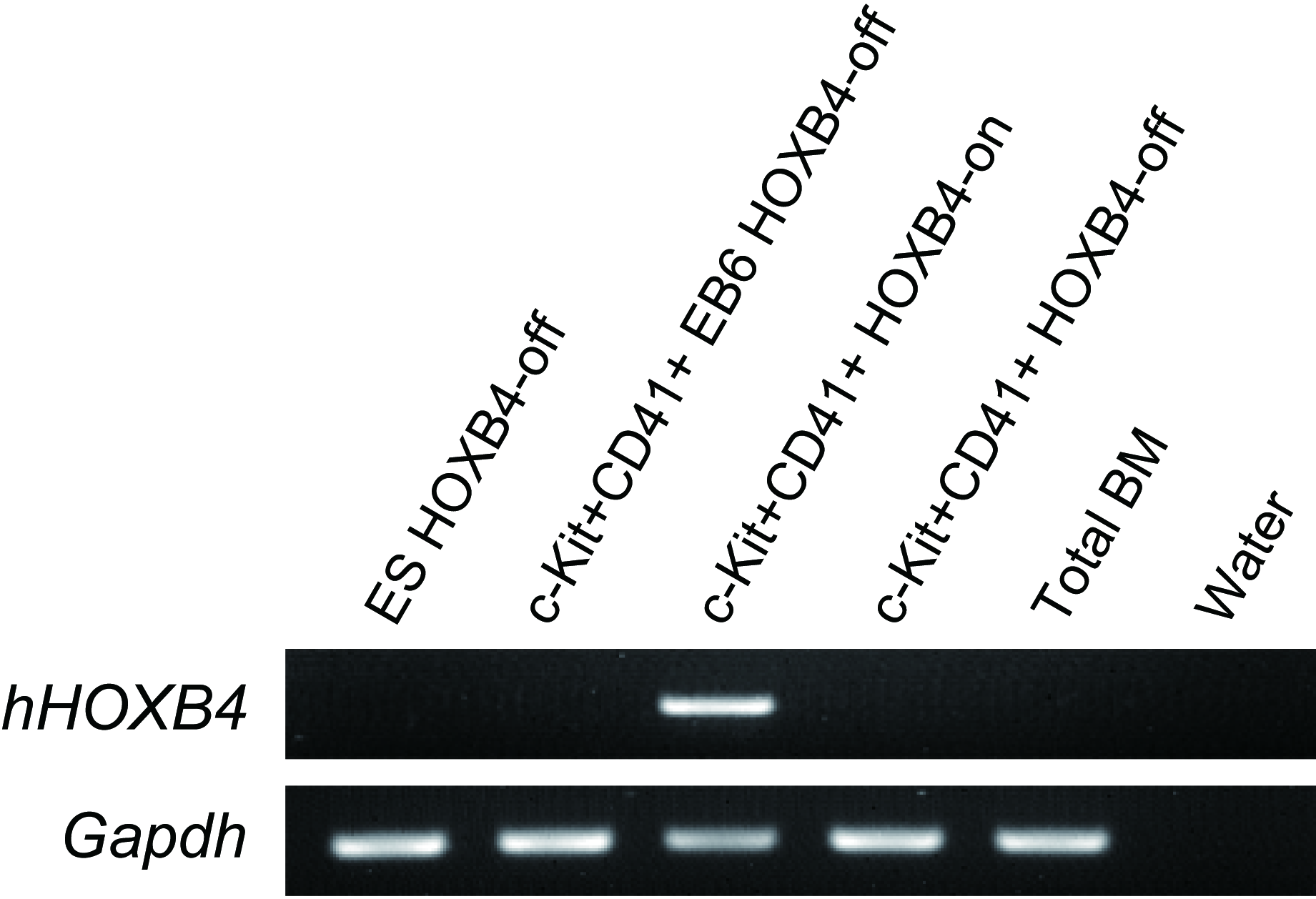

Supplement: Figure S5 — RT-PCR analysis for induced HOXB4 expression in c-Kit+CD41+ cells. ESCs were maintained in the presence of Dox (ES HOXB4-off). After ESCs were differentiated into EB6 cells in the presence of Dox, c-Kit+CD41+ cells were isolated (c-Kit+CD41+ EB6 HOXB4-off). These cells were co-cultured with OP9 cells in the presence or absence of Dox for 4 days, followed by recovery of c-Kit+CD41+ cells from the co-cultures (c-Kit+CD41+ HOXB4-off and c-Kit+CD41+ HOXB4-on). These c-Kit+CD41+ cells and ESCs along with adult bone marrow cells (Total BM) as a negative control were examined for HOXB4 expression by RT-PCR analysis. The PCR program consisted of 15 sec at 95°C, 15 sec at 60°C, and 30 sec at 72°C. A total of 42 cycles or 30 cycles was used for amplification of HOXB4 or Gapdh. (9.28 MB TIF) [file pone.0004820.s010.tif]

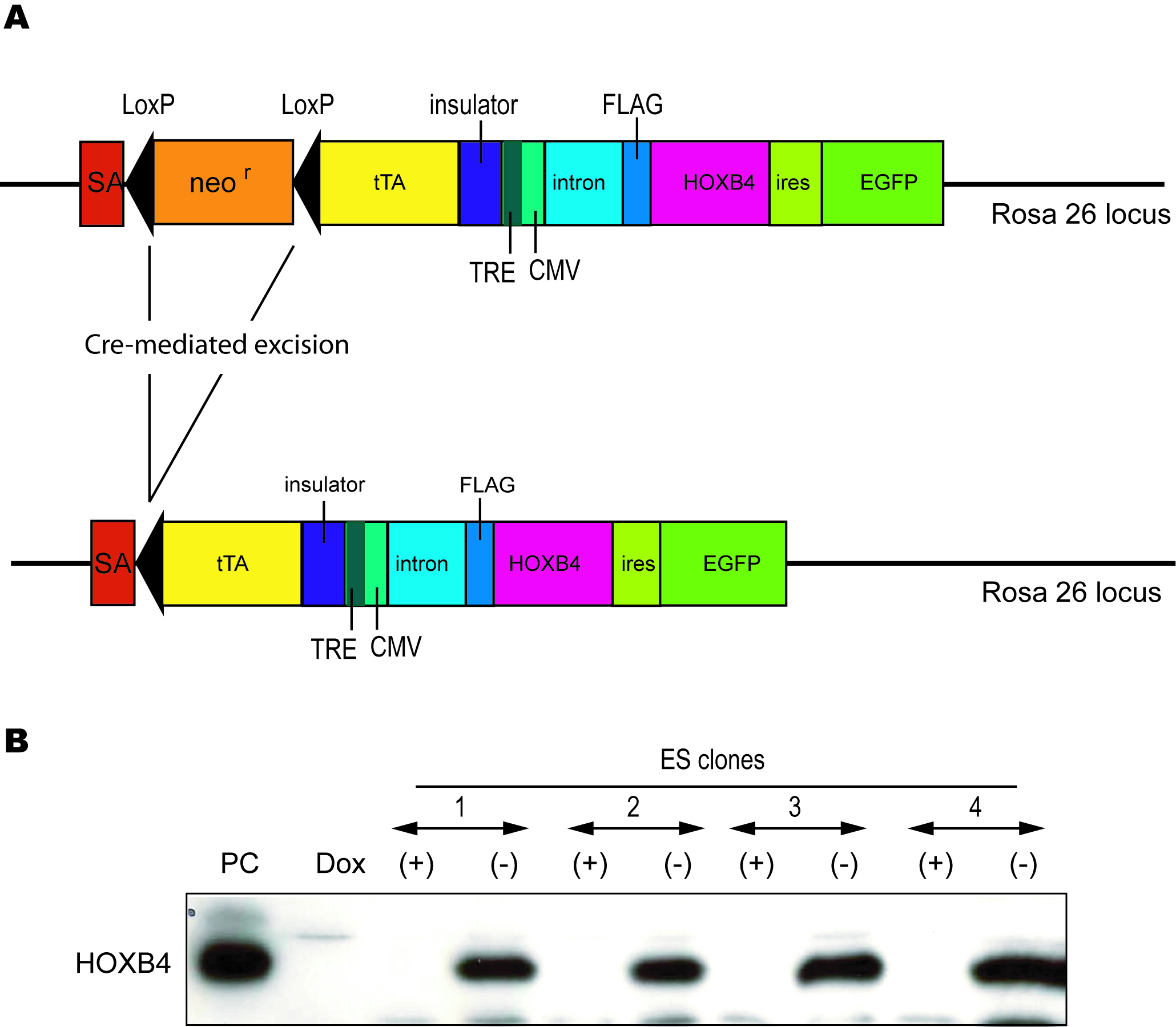

Supplement: Figure S6 — Tet-off inducible HOXB4/EGFP expression system. (A) Schematic presentation of the Tet-off HOXB4 gene expression cassette integrated into the constitutive active ROSA 26 locus on chromosome 6. HOXB4 cDNA was kindly provided by Dr. K. Humphries (Terry Fox Laboratory, Vancouver, Canada). The Tet-off regulated gene expression plasmid comprised a splice-acceptor (SA) sequence; a loxP-flanked neomycin phosphotransferase gene (neor) gene, including a polyA signal; the Tet-controlled transcriptional activator (tTA) gene, including a polyA signal; an insulator sequence; the tTA-responsive element (TRE), followed by the minimal immediate-early promoter from Cytomegalovirus (CMV); the rabbit beta-globin 2nd intron; human HOXB4 cDNA; an internal ribosome entry site (ires); EGFP cDNA; and a polyA signal. The constructed vector was amplified in E. coli Stabl2 cells (Invitrogen), purified using a GENOPURE plasmid maxi kit (Roche), linearized by SwaI digestion, and used to transfect ESCs. The Tet-regulated HOXB4/EGFP expression cassette was integrated into the constitutively active ROSA26 locus in EB3 cells by homologous recombination. In brief, EB3 ESCs were electroporated with the linearized vector and were selected with G418 (150–200 µg/ml). G418-resistant colonies were picked and ES clones carrying a targeted integration of the vector in the ROSA26 locus were identified by long distance-PCR analysis using the following primers: forward (ROSA26 locus 1st exon), 5′-CCTCGGCTAGGTAGGGGATCGGGACTCT-3′; reverse (neor gene), 5′-CGGAGAACCTGCGTGCAATCCATCTTGTTC-3′; forward (EGFP), 5′-GGATCACTCTCGGCATGGACGAGCTGTAC-3′; and reverse (ROSA26 locus 2nd exon), 5′-AGCCTTAAACAAGCACTGTCCTGTCCTCAAG-3′. The PCR cycles consisted of one cycle at 94°C for 1 min, 32 cycles at 98°C for 20 s, 66°C for 30 s, 68°C for 4 min, and one cycle at 72°C for 10 min. To remove the loxP-flanked neor gene, Cre recombinase was transiently expressed in the selected clones by transfection with the pCAG-cre-IRES-puro plasmi [file pone.0004820.s011.tif]
